# *Caenorhabditis elegans* hub genes that respond to amyloid beta are homologs of genes involved in human Alzheimer’s disease

**DOI:** 10.1371/journal.pone.0219486

**Published:** 2019-07-10

**Authors:** Rasoul Godini, Roger Pocock, Hossein Fallahi

**Affiliations:** 1 Department of Biology, School of Sciences, Razi University, Kermanshah, Iran; 2 Development and Stem Cells Program, Monash Biomedicine Discovery Institute and Department of Anatomy and Developmental Biology, Monash University, Melbourne, Australia; H Lee Moffitt Cancer Center and Research Institute, UNITED STATES

## Abstract

The prominent characteristic of Alzheimer’s disease (AD) is the accumulation of amyloid beta (Abeta) proteins in the form of plaques that cause molecular and cellular alterations in the brain. Due to the paucity of brain samples of early-stage Abeta aggregation, animal models have been developed to study early events in AD. *Caenorhabditis elegans* is a genetically tractable animal model for AD. Here, we used transcriptomic data, network-based protein-protein interactions and weighted gene co-expression network analysis (WGCNA), to detect modules and their gene ontology in response to Abeta aggregation in *C*. *elegans*. Additionally, hub genes and their orthologues in human and mouse were identified to study their relation to AD. We also found several transcription factors (TFs) responding to Abeta accumulation. Our results show that Abeta expression in *C*. *elegans* relates to general processes such as molting cycle, locomotion, and larval development plus AD-associated processes, including protein phosphorylation, and G-protein coupled receptor-regulated pathways. We reveal that many hub genes and TFs including *ttbk-2*, *daf-16*, and *unc-49* have human and mouse orthologues that are directly or potentially associated with AD and neural development. In conclusion, using systems biology we identified important genes and biological processes in *C*. *elegans* that respond to Abeta aggregation, which could be used as potential diagnostic or therapeutic targets. In addition, because of evolutionary relationship to AD in human, we suggest that *C*. *elegans* is a useful model for studying early molecular events in AD.

## Introduction

Extracellular senile plaques and neurofibrillary tangles of tau protein are the main hallmarks of Alzheimer’s disease (AD). Senile plaques are composed of amyloid beta (Abeta) peptides with neurotoxic features that lead to synaptic dysfunction, connectome disruption, and neural death. In familial AD, Abeta deposition is related to mutations in either Abeta precursor protein (APP) or catalytic subunits of γ-secretase, presenilin-1 (PS1) or presenilin-2 (PS2) [1]. γ-secretase and β-secretase are membrane-bound proteases that cleave APP to Abeta. γ-secretase cleaves the C-terminal fragment of APP and generates multiple types of Abeta, based on the position of cleavage [2]. Two dominant forms of Abeta40 and Abeta42 constitute approximately 80–90% and 5–10% of total Abeta in normal cells, respectively. However, Abeta42 is the major molecule in the formation of Abeta plaques, which is observed in one-third of plaques [3]. Abeta peptides are involved in multiple physiological processes, including regulation of synaptic activity, transcription, neuronal survival, processing of APP, and antioxidant activity [4–6]. Removal of Abeta from cells is achieved via neprilysin (insulin-degrading enzyme) followed by lysosomal degradation (REF). It can also be transported into blood vessels and carried away for degradation [1]. Despite the importance of these peptides in AD, it is not possible to monitor the accumulation trends and its impact on human neural cells. Therefore, the role of Abeta and its accumulation is analyzed in animal model systems of AD.

Transgenic modifications have generated models of AD in animals including primates [7], mouse [8], rat [7], fruit fly [9], and nematode (*Caenorhabditis elegans*) [10]. Although *C*. *elegans* is a convenient model for neurodegenerative disease, it differs from humans in lacking Abeta and β-secretase [10]. These animals do not exhibit human pathological and behavioral symptoms of AD, but they can be used for studying molecular mechanisms related to Abeta aggregation. Using transgenic techniques, several *C*. *elegans* strains have been generated to express human Abeta, where Abeta precipitates in muscle and leads to paralysis [10]. To study early responses to Abeta aggregation and to identify proteins and pathways affected by Abeta, model systems have been introduced that harbor temperature-dependent induction of Abeta expression [11]. In such models, the gene expression profiles can be studied using high-throughput techniques at the onset of Abeta accumulation as described in the upcoming sections.

Transcriptome data can also be analyzed using network approaches to determine modules of genes or proteins, molecular mechanisms, interactions between members of a system, and the most important proteins in a pathway that could be used as potential candidates for further experiments [12]. Weighted Gene Co-expression Network Analysis (WGCNA) is a robust method, widely used to detect important members (genes) and modules of genes that permit downstream functional analysis [13]. WGCNA estimates the pair-wise correlation between genes, using expression values, and identifies groups of genes with a similar pattern of expression [14]. Another informative network analysis method is the Protein-Protein Interaction (PPI) network approach. Proteins can function in complexes, therefore studying these interactions is effective in identifying key members in networks. Information to construct such networks originates from either experimental findings or *in silico* predictions [15].

Here, we have applied a systems biology approach, using data from transgenic *C*. *elegans* models of AD that synthesize Abeta protein, to find early molecular responses and potentially important genes and proteins in the response to Abeta. Several studies have used AD biomarkers that show the onset and progress of the disease, however, they are still not able to determine molecular modifications in brain tissue at early stages [16]. We have detected orthologous genes in worm, mouse and human suggesting that similar pathways are affected by Abeta expression in these evolutionarily distant species.

## Material and methods

### Microarray data description

**Fig 1:**
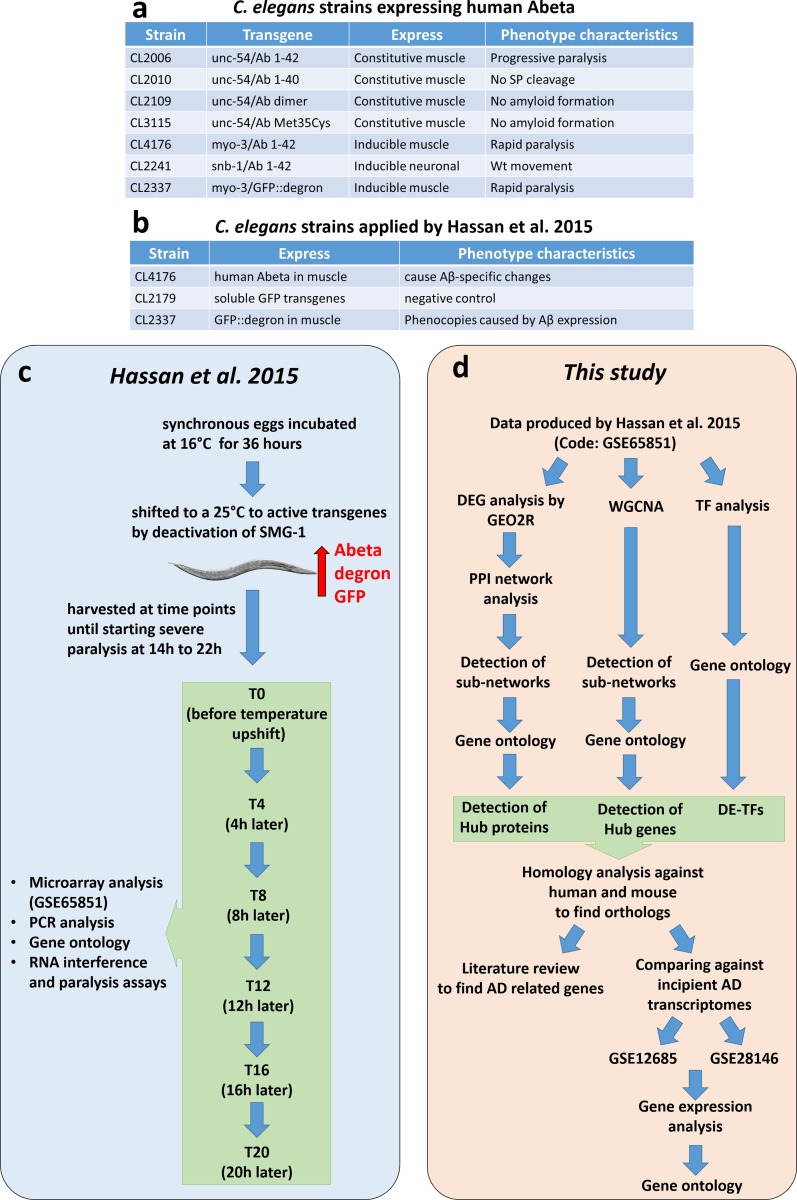
Comparing methodology of original paper and present study and Abeta expressing *C*. *elegans* transgenic strains. a) The model expresses human Abeta that results in alteration phenotypes (please refer to Material and Methods section), b) *C*. *elegans* strains used by Hassan and colleagues, c) Experimental design and methodology by Hassan and colleagues. Briefly, synchronized eggs of transgenic worms were grown at 16˚C for 36 h and shifted to 25˚C to activate the *smg-1* gene. Worm samples were collected at 4 hours intervals from T0 (temperature shift time) to T20 (before worm paralysis), and analysis including microarray, PCR, gene ontology, and RNA interference were performed in the original study. d) Pipelines used in this study to analyze publicly available datasets GSE65851, and datasets for incipient AD including GSE12685 and GSE28146.

In order to study the early effects of Abeta on *C*. *elegans* at the systems level, we searched the Gene Expression Omnibus (GEO) database of National Center for Biotechnology Research (www.ncbi.nlm.nih.gov/gds) using “amyloid beta” keyword and filtered the outcome by selecting *C*. *elegans* as the organism. We found that among all resulting datasets only one study under accession number GSE65851, originally published by Hassan and colleagues 2015, would be useful for pursuing our goal (having a sufficient sample number and experimental design within early stages and including a successive time-course). In this study, Abeta toxicity was investigated in *C*. *elegans*, using a strain (CL4176) that expresses human Abeta42 in body wall muscle. Toxicity was examined at 4-hour intervals from time-point 0 to time-point 6 (20 hours) [11]. This dataset contains 47 microarrays, 15 of which are related to Abeta toxicity and the remaining are related to GFP expression, as the negative control, or GFP-degron to study the impact of Abeta protein aggregation on cells [11]. Briefly, they performed microarray analysis, gene ontology RNA interference, and paralysis assays on samples collected from several time-points with 4-hour intervals. The sample collection covered time points between T0 and T20, where severe paralysis begins. Through these time points the process of early response to Abeta could be studied. Here, we performed network-based analysis and gene ontology to find relationships between genes and potential hub genes (Fig 1). Furthermore, to independently validate our findings, two additional microarray datasets from the human brain were also analyzed. These two datasets were GSE12685 that encompass 6 AD and 8 control samples from the original study by Williams and colleagues 2009 [17], and GSE28146 containing 7 incipient AD and 8 control arrays published by Blalock et al. 2011 [18].

### Data preparation and gene expression analysis

To obtain DEGs (Differentially expressed genes) between different time-points and experimental conditions, GEO2R tool (www.ncbi.nlm.nih.gov/geo/geo2r/) was used, which is embedded in NCBI. For the Abeta study in worms, two sets of analysis were performed. First, the transcriptome profiles of Abeta-manipulated samples were compared to that of GFP expressing samples. While in the second analysis, the expressions of genes were studied in samples over consecutive time-points. Besides, at each time point, the gene expression of GFP samples were removed from that of Abeta accumulation to exclude developmentally linked DEGs. Significantly affected genes were identified by applying two filters; first, selecting only those genes with a |log2 FC| of 0.6 between two conditions and then a p-value ≤ 0.005 (for the time-course study) or p-value ≤ 0.01 (for the comparing made between Abeta and GFP samples). Similarly, in datasets that were used for independent validation, genes harboring a p-value ≤ 0.05 and showing ±0.6 changes in Log2FC were considered as differentially expressed genes (DEGs). Next, all of the probes without annotation were removed. For duplicate probes, those with the lowest p-values were included in the analysis. To prepare the required high-quality input matrix for the WGCNA package, one needs to determine the distribution of the samples and data quality for network analysis. To achieve this, we first performed principal component analysis (PCA) and filtered outliers. To further eliminate bias and noise from the data, the matrix of gene expression normalized by log2 transformation and probes with average log2 expression value less than 0.6 (through all arrays) were discarded. Based on the coefficient of variations, only 0.9 percentile of the genes were selected as input for WGCNA.

### Gene coexpression analysis via WGCNA

WGCNA analysis was performed according to Langfelder and Horvath, 2008 [14]. Applying R package WGCNA, we constructed an “unsigned” network for all arrays using “soft-power = 12”, and “deep-split = 2”. The remaining default thresholds were used with no further changes. Module eigengenes were obtained and their relationships with different time-points were determined. At each time-point, highly related modules were selected to determine the correlation between module membership and gene significance. Then modules with the highest correlation were identified and used for further analysis. Networks were visualized and analyzed using either Cytoscape 3.4.0 [19] or Gephi 0.9.2 [20].

### PPIs network among differentially expressed gene products

Several “undirected” PPI networks were constructed using DEGs and interactions between proteins were extracted from the STRING database [21]. This was achieved by submitting DEG lists to the database and selecting information for *C*. *elegans*. Interactions were filtered for being “experimentally validated”, “information obtained from other databases”, and “co-expression”, and meet a medium confidence threshold of 0.4. PPI networks were analyzed and visualized by Cytoscape 3.4.0 [19] and Gephi 0.9.2 [20]. To detect protein complexes as sub-networks, we used ClusterONE plugin in Cytoscape software [22], considering the following settings: minimum size: 5; minimum density: Auto; Edge weight: string confidence value. All the presented modules had p-values ≤ 0.005. The top 10 percent of nodes with the highest weighted degree were selected as hub genes in the PPI network.

### Transcription factor analysis

The list of TFs in *C*. *elegans* was retrieved from Reece-Hoyes and colleagues study [23]. They identified a set of 934 TFs by computational analysis and manual curation. Here, using hierarchical clustering and correlation method, we clustered the TFs in our datasets based on their log2 normalized expression values through all arrays. Additionally, differentially expressed TFs (DE-TFs) in at least two stages or overexpressing DE-TFs (see the results section) were selected and clustered. The clustering was performed by Heatmap.3 in R.

### Gene ontology and enrichment map construction

We used the Database for Annotation, Visualization and Integrated Discovery (DAVID) 6.8 for gene ontology analysis [24]. For each gene list, we have selected up to 5 biological process terms with the lowest p-value (≤ 0.05). For constructing an enrichment map of biological processes we have used Enrichment map 3.0.0 plugin of Cytoscape, using default settings [25].

### Homology analysis and literature review

To identify human and mouse orthologous genes for the hub genes in *C*. *elegans*, “tblastn” was used on the NCBI server (blast.ncbi.nlm.nih.gov/Blast.cgi). First, FASTA format of the longest protein sequences were fetched for the selected genes and submitted to “tblastn” and blasted against *Homo sapiens* and *Mus musculus* using default parameters. Then, the top hits with E-values below 1e-10 were obtained and considered as the human or mouse counterparts of the input genes.

A literature review was performed by searching “Google” (www.google.com), “Google Scholar” (www.scholar.google.com), and “PubMed” (www.ncbi.nlm.nih.gov/pubmed) with keywords, including “[the gene name]” with either “Alzheimer”, “Abeta”, or “Amyloid Beta” terms. This approach helped us to find potential publications directly related to the genes of interest. Next, the resources were manually checked to determine the role of the given gene in AD (if any). In doing so, we have determined possible changes in gene expression, methylation, and protein level alterations in addition to single nucleotide polymorphism in the gene of interest in AD. Consequently, a relationship between the gene of interest and AD or any other disorders in neuron activity was established (direct or indirect). We also conducted similar approaches for gene aliases.

## Results

### Data selection

Here, we have analyzed transcriptome data produced by Hassan and colleagues in which they used a transgenic *C*. *elegans* model expressing Abeta in body wall muscle [11]. It is known that *C*. *elegans* is a suitable model to study neurodegenerative diseases including AD [26].

### PCA and DEGs analysis

**Fig 2:**
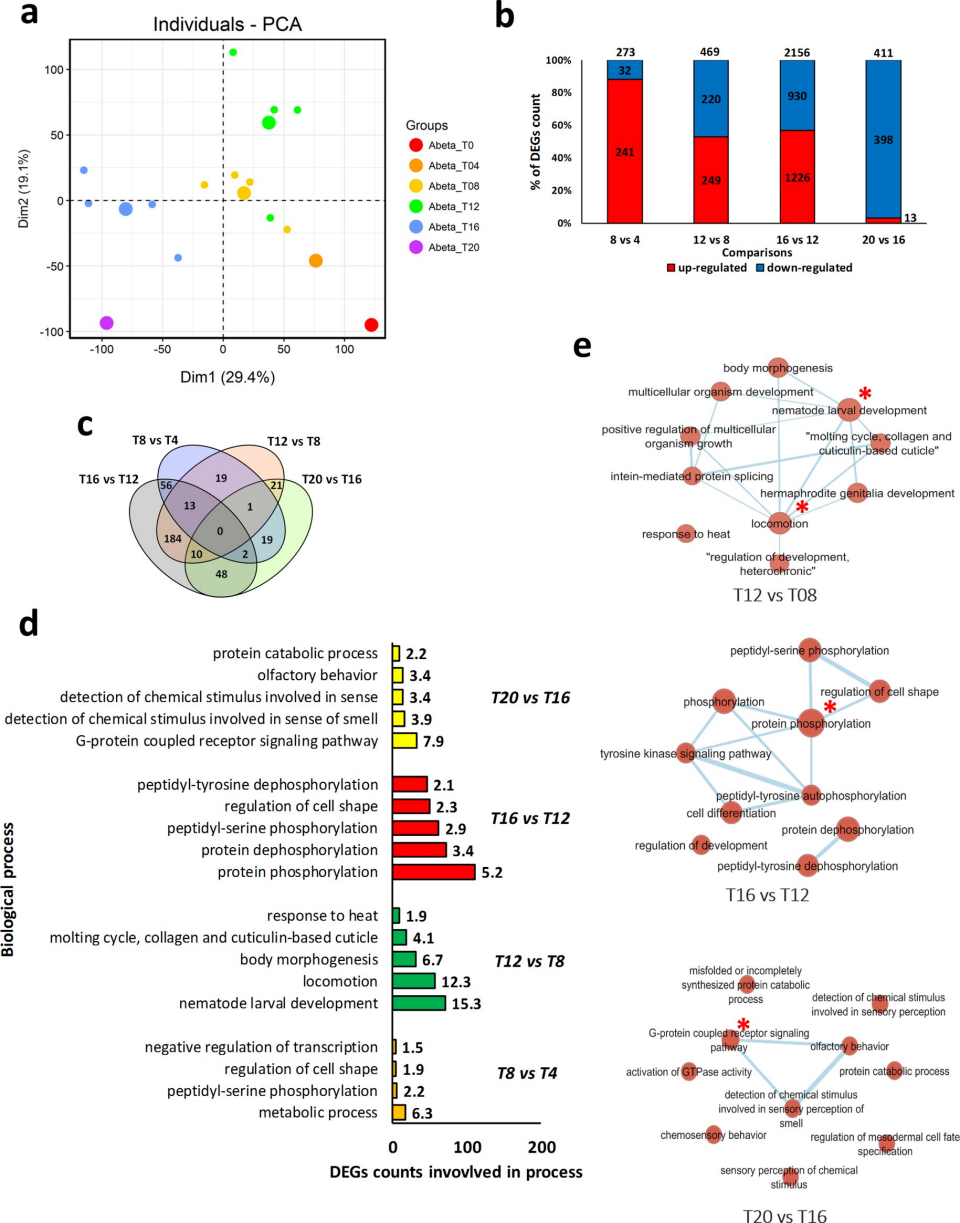
*C*. *elegans* transcriptome and gene ontology enrichment analyses. a) Principle Component Analysis (PCA) of the samples. b) A bar graph for the number of differentially expressed genes at each time-point and the number of up and down-regulated genes. Maximum deregulation of genes occurred at transition from T12 to T16. c) A Venn diagram for the comparison of the DEGs among time-points after Abeta expression. d) Affected biological processes in each time-point comparison. The numbers close to the bars indicate the percentage of DEGs that are involved in each process. e) Enrichment map of biological processes, in which nodes are biological processes and edges show common genes between two nodes. Larger nodes represent those processes that contain more DEGs. Main process in each network is indicated by red star. There was no enrichment network for the T8 *vs*. T4 comparison. In PCA graph large dots are average of all the sample of that group.

We performed PCA analysis to detect the distribution pattern and to identify any potential outliers of the microarray samples containing gene expression data of Abeta-expressing worms at different time-points [12]. The samples were collected at 4-hour intervals from time-point 0 to 6, thereafter denoted as T0, T4, T8, T12, T16, and T20. Our analysis show that the samples of each time-point are clustered with minor overlaps between T8 and T12 (Fig 2A). Notably, only one repeat in T0, T4, and T20 failed to group with their counterpart samples. As the samples were grouped as expected (no outlier was found), all samples were included in the remainder of our analysis. Next, DEGs were identified using GEO2R, a tool embedded in NCBI, that uses R package Limma. As this tool requires at least two samples in each treatment, we were not able to compare the gene expression pattern of time-points T0 and T4. Analyzing the other time-points, we detected the highest number of DEGs in the comparison between time-points T16 and T12 (with 2156 DEGs), which is in agreement with the PCA result. While the lowest number of DEGs (273 DEGs) was detected for the comparison made for the time-point T8 *vs*. T4. The highest differences between up- and down-regulated DEGs observed in the comparison of T8 *vs*. T4 (with 241 and 32 genes being up- and down-regulated, respectively), and in the results obtained for the T20 *vs*. T16 comparison (where 13 and 398 genes were up- and down-regulated, respectively). In two other comparisons, namely T16 *vs*. T12 and T12 *vs*. T8, an almost equal number of up- and down-regulated genes were observed (Fig 2B). Venn diagram representation was used to show the common DEGs among different comparisons (Fig 2C). The highest number of common DEGs were found between the comparisons made for time-points T16 *vs*. T12 stage and T12 vs. T8, with 184 DEGs. Only 13 DEGs were found in all comparisons including T16 *vs*. T12, T12 *vs*. T8, and T8 *vs*. T4. Interestingly, no genes were present in all time-points, indicating time-point specific expression trends.

Gene ontology enrichment analysis showed that different biological processes are associated with the different stages of transition (Fig 2D). Metabolic processes (6.3% of DEGs involved in this processes) and cell shape regulation (1.9% of DEGs) are the most affected processes at the transition from T4 to T8 (named as stage one). While, during the transition from T8 to T12 (stage two), processes including nematode larval development (15.3%), locomotion (12.7%), and body morphogenesis (6.7%) are represented by the highest number of DEGs. Protein phosphorylation was dominant process at the transition from T12 to T16 (stage three), however, cell shape regulation (2.3% of DEGs) was also observed. Annotation of DEGs in the T16 to T20 (stage four) transition indicated that G-protein coupled receptor signaling pathway (7.9% of DEGs), and detection of chemical stimuli involved in sensing (3.9% of DEGs) were affected the most. Furthermore, enrichment map analysis was performed to detect connections between these processes and to identify the most central processes at each transition (Fig 2E). In the second stage, locomotion and nematode larval development were the most important processes. While, protein phosphorylation and G-protein coupled receptor signaling pathways were the most central processes in the third and fourth stages, respectively. No enrichment map was detected for the first stage.

**Fig 3:**
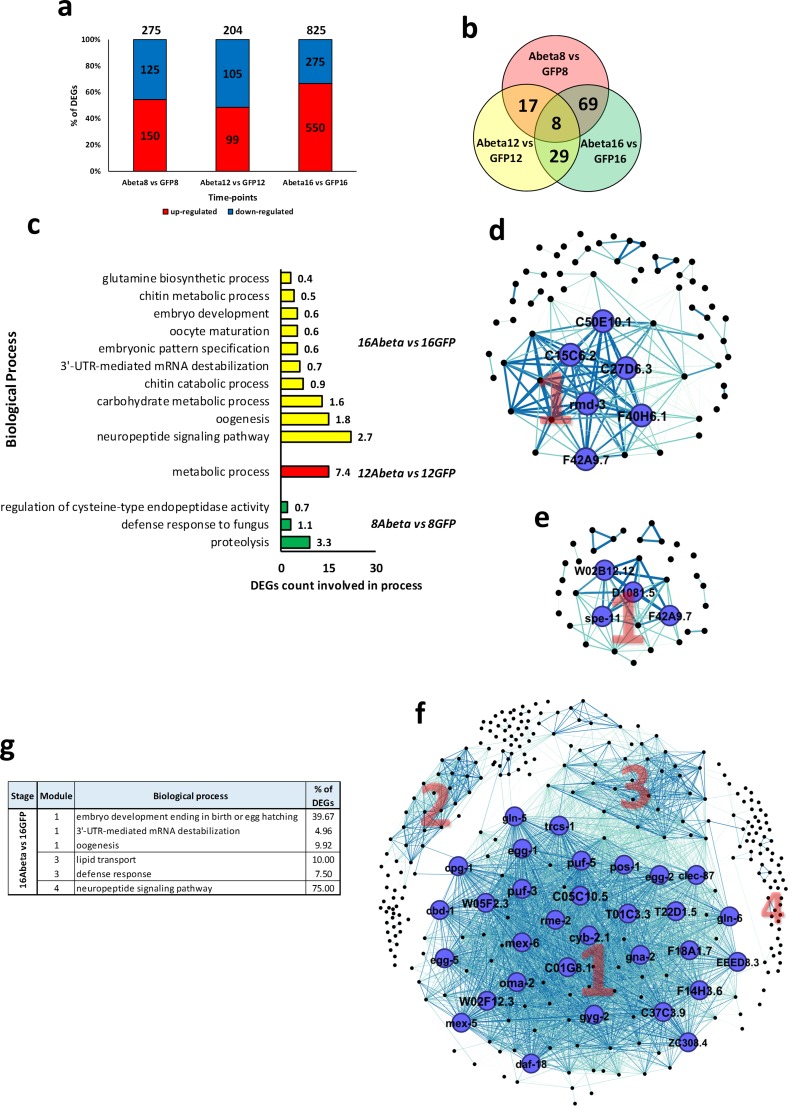
*C*. *elegans* transcriptome analysis of Abeta versus GFP samples, PPI networks, and gene ontology enrichment. a) DEGs in each time-point. b) Venn diagram of common DEGs among time-points. c) Bar graph of biological processes that DEGs are involved in. d) PPI network of T8. e) PPI network of T12. f) PPI network of T16. g) Table of top three biological processes in the modules from networks. Note—the biological processes with P-value less than 0.05 were selected. In the network, modules are indicated by numbers. Nodes and labels in larger size indicate higher centrality-based degree factor. Thicker edges indicate higher weight. There was no network for time-points T8 and T12.

When Abeta and GFP samples were compared (Fig 3), we found a lower number of DEGs compared to the time-points comparisons. Here, the highest number of affected genes were at time-point T16, where 825 DEGs were detected (Fig 3A and 3B). Gene ontology indicates that proteolysis, metabolic processes, and neuropeptide signaling pathways are important biological processes associated with these DEGs (Fig 3C).

### PPI network analysis

**Fig 4:**
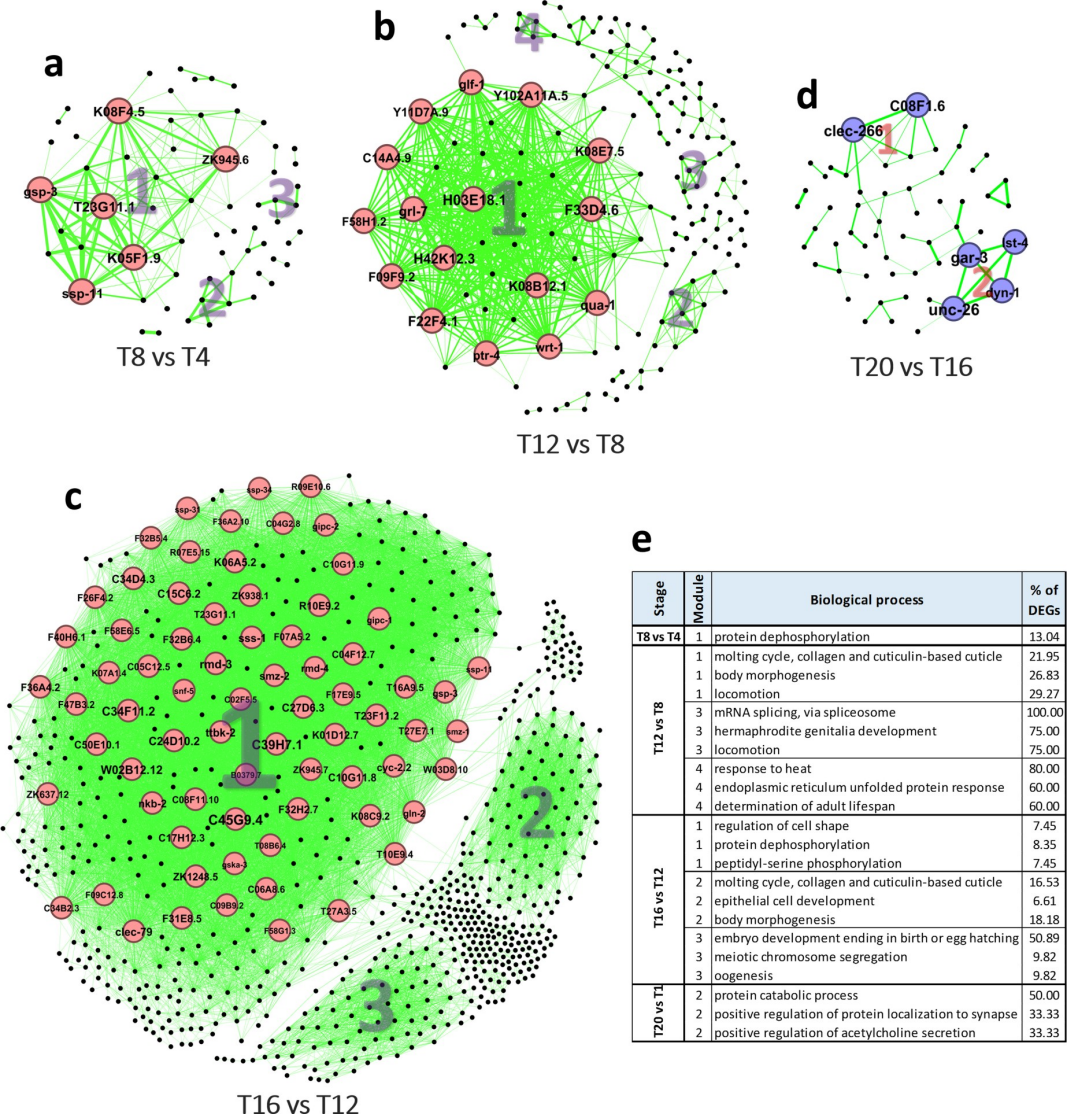
PPI networks and gene ontology of module members obtained for each network. a) PPI network of T8 *vs*. T4 stage. b) PPI network of T12 *vs*. T8 stage. c) PPI network of T16 *vs*. T12 stage. d) PPI network of T20 *vs*. T16 stage. e) Table contains information regarding the top three biological processes found for the genes in the modules detected in each network. The biological processes with p-value less than 0.05 were selected. In the network, modules are indicated by numbers. Red and blue colors of nodes represent up- and down-regulation, respectively. Nodes and labels in larger size indicate higher centrality based on degree of connectivity factor. Thicker edges indicate higher weight.

PPI networks were constructed for each stage using available information obtained from the STRING database. Then, sub-networks of complexes and highly connected central proteins were detected. In addition, using the gene ontology of sub-networks we identified underlying biological processes. PPI networks constructed for the first stage (T8 *vs*. T4) had 3 sub-networks, where only module one was significantly linked to an important process, being protein de-phosphorylation (Fig 4E). Six hub genes *K05F1*.*9*, *T23G11*.*1*, *K08F4*.*5*, *ssp-11*, *gsp-3*, and *ZK945*.*6* were found as top 10% genes with high centrality, which all were up-regulated (Fig 4A). In the second stage (T12 *vs*. T8) there were four sub-networks relating to molting cycle, body morphogenesis, mRNA splicing, and response to heat stress (Fig 4E). At this stage, we detected 16 hub genes including *H03E18*.*1*, *F33D4*.*6*, *H42K12*.*3*, *F22F4*.*1*, *grl-7*, *qua-1*, *K08B12*.*1*, *Y102A11A*.*5*, *K08E7*.*5*, and *F09F9*.*2*, all that were up-regulated (Fig 4B). PPI network of the third stage (T16 *vs*. T12) had three sub-networks relating to regulation of cell shape, protein dephosphorylation, molting cycle, and embryo development (Fig 4E). Altogether, 72 hub genes were detected that all were up-regulated at this stage. Among them, we detected *C45G9*.*4*, *C39H7*.*1*, *C34F11*.*2*, *rmd-3*, *W02B12*.*12*, *C24D10*.*2*, *ttbk-2*, *clec-79*, *C34D4*.*3*, and *C15C6*.*2* as the top 10 most central genes (Fig 4C). In the final stage (T20 *vs*. T16), the PPI network had 2 sub-networks, where module 2 was related to protein catabolic processes, and positive regulation of protein localization to synapse (Fig 4E). There were six down-regulated hub genes, including *unc-26*, *gar-3*, *clec-266*, *C08F1*.*6*, *lst-4*, and *dyn-1*, which were present in both sub-networks (Fig 4D). We also constructed PPI for Abeta *vs*. GFP comparisons (Fig 3). Although subnetworks were determined in all networks, only those in T16 had statistically significant functional annotations, including embryo development, lipid transport, and neuropeptide signaling pathway (Fig 3G).

### WGCNA construction and module detection

**Fig 5:**
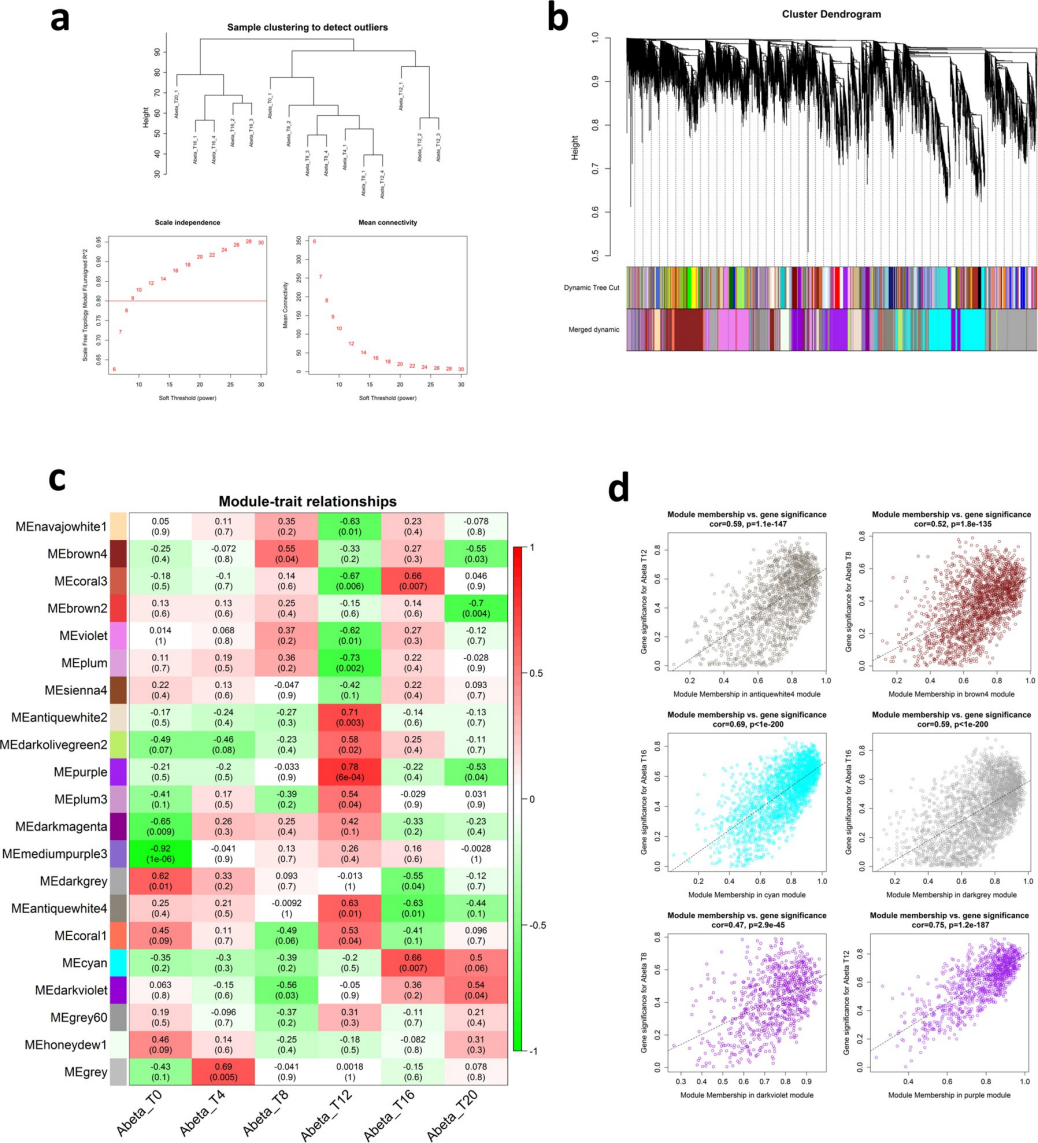
Graphs produced by WGCNA. a) Hierarchical clustering of the samples and soft-power detection. The graph indicates that soft-power above 10 meets scale free topology above 0.8. b) Module detection and merging modules with 75 similarities in eigengenes. c) Module-trait relationship heatmap. Only those modules with high relationships (0.3<) were selected for further analysis. d) Module membership *vs*. gene significance of the highly correlated modules with specific time-points. Only modules with p-value ≤ 1e-5 were considered for further analysis.

**Fig 6:**
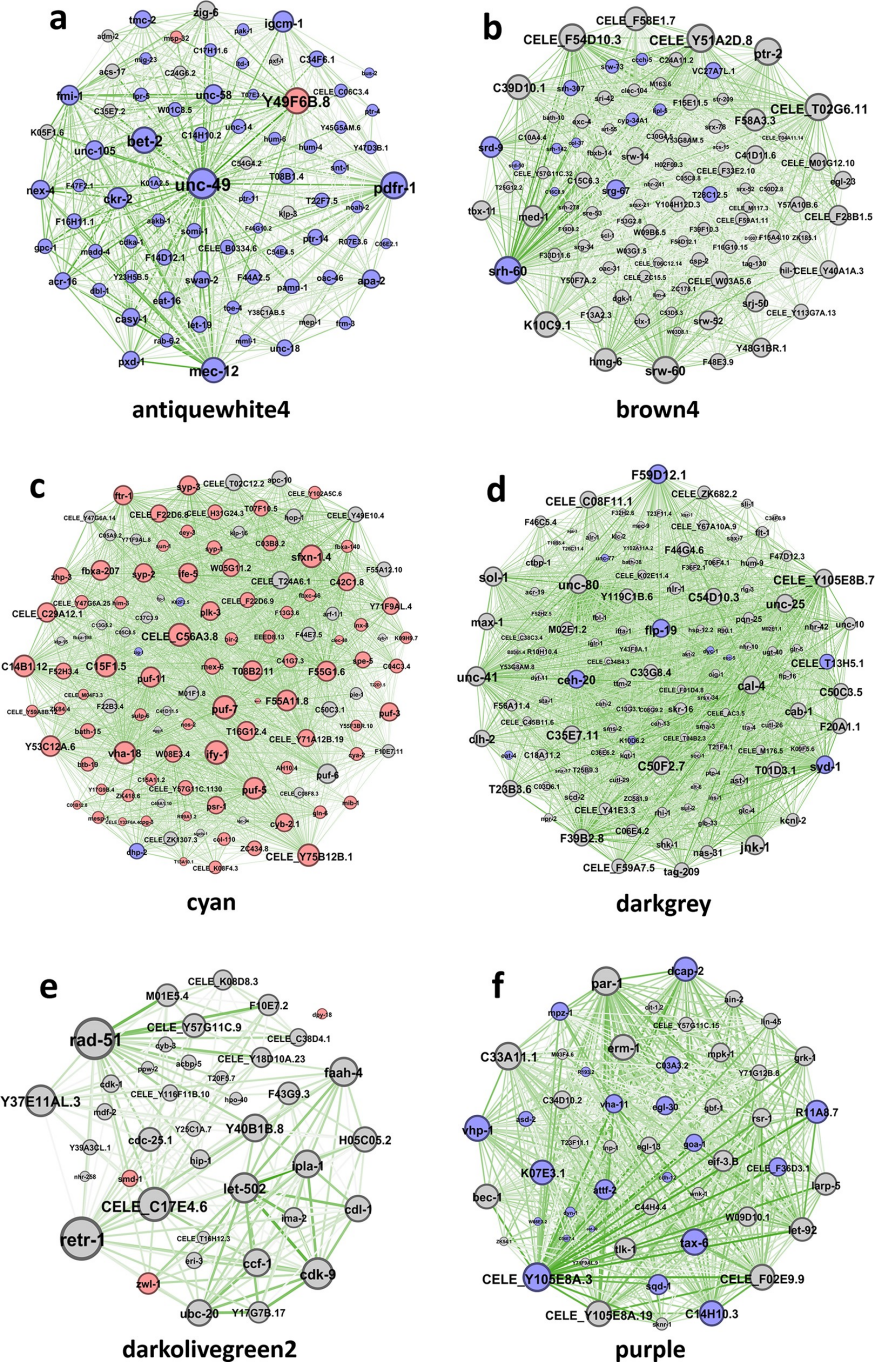
Networks of the top 10 percent of most central genes in selected modules identified by WGCNA. Identified modules were labeled as a) “antiquewhite4”, b) “brown4”, c) “cyan”, d) “darkgrey”, e) “darkolivegreen2”, and f) “purple”. Red and blue colors of nodes represent up- and down-regulation, respectively. Grey nodes represent no DEGs for that gene. Nodes and labels in larger size indicate higher centrality in the network based on degree factor. Thicker edges indicate higher weight in terms of network analysis. Only fifty percent of edges are shown.

We have detected modules and highly connected genes related to each time-point by applying WGCNA to the list of gene expression values. Hierarchical clustering grouped the time-points together, with some exceptions. No outliers were detected in the samples, which is in agreement with the PCA results. Soft-threshold analysis revealed that soft-power 12 would be the best value for module detection (Fig 5A). Following network construction and module detection, module-time-point relationships were also investigated (Fig 5B and 5C). In total, 8 out of 21 detected modules were mostly related to three time-points T8, T16, and particularly T12. To show exact relationships with these time-points, module membership *vs*. gene significance correlation analysis was performed for the most related modules (Fig 5D). We selected the top 8 modules that were highly correlated with at least with one of the time-points. It should be mentioned that some modules, in spite of being highly correlated, did not have enough members to pass the significance threshold for gene ontology analysis. Altogether, modules named as “antique white 4”, “brown 4”, “cyan”, “dark grey”, “dark olive green 2”, “dark violet”, “purple”, and “violet” were identified and gene ontology enrichment was performed for them. Several important biological processes were affected by the expression of Abeta protein at different time-points, including G-protein coupled receptor signaling pathway, transport, regulation of transcription, meiotic nuclear division, embryo development, locomotion, and reproduction (Table 1). In order to reduce the number of the modules and increase the significance of the results, the modules with similar gene ontology and lower correlation with the time-points were removed and only those with highest significant in GO were considered for further analysis. Finally, six modules including “antique white 4”, “brown 4”, “cyan”, “dark grey”, “dark olive green 2”, and “purple” were selected for network analysis and to detect the hub genes in response to Abeta protein. Interestingly, we found that many of the hub genes in the modules identified as “antiquewhite4”, “cyan”, and “purple” were differentially expressed. Expression patterns of these hub DEGs were similar among all modules, where the genes were down-regulated in modules labeled as “antiquewhite4”, and “purple”, whereas they were up-regulated in the module named “cyan” (Fig 6).

**Table 1:** Top five biological processes with lowest p-value for detected modules by WGCNA.

Modules	Term	Count	% of ME members	P-Value
**antiquewhite4**	molting cycle, collagen and cuticulin-based cuticle	52	3.97	2.33E-08
	transport	101	7.72	3.02E-07
	positive regulation of multicellular organism growth	54	4.13	1.12E-06
	ion transport	51	3.90	1.52E-05
	determination of adult lifespan	108	8.25	2.94E-05
**brown4**	G-protein coupled receptor signaling pathway	114	7.42	8.74E-19
	detection of chemical stimulus involved in sensory perception of smell	61	3.97	7.34E-17
	olfactory behavior	63	4.10	1.27E-16
	detection of chemical stimulus involved in sensory perception	61	3.97	5.55E-12
	sensory perception of chemical stimulus	33	2.15	3.48E-06
**cyan**	meiotic chromosome segregation	39	2.16	4.22E-10
	meiotic nuclear division	32	1.77	1.81E-08
	kinetochore assembly	7	0.39	1.53E-05
	cell cycle	30	1.66	2.98E-05
	synaptonemal complex assembly	6	0.33	3.85E-05
**darkgrey**	regulation of transcription, DNA-templated	160	7.48	1.02E-14
	ion transport	93	4.35	6.04E-14
	steroid hormone mediated signaling pathway	78	3.64	1.50E-13
	transcription, DNA-templated	126	5.89	8.00E-13
	transport	155	7.24	1.02E-10
**darkolivegreen2**	embryo development ending in birth or egg hatching	82	26.20	7.14E-05
	mitotic nuclear division	10	3.19	2.55E-04
	reproduction	61	19.49	3.73E-04
	cell division	10	3.19	8.12E-04
	proteasome-mediated ubiquitin-dependent protein catabolic process	6	1.92	0.001218
**darkviolet**	cellular response to DNA damage stimulus	24	3.79	6.85E-10
	DNA repair	22	3.47	5.65E-09
	nucleic acid phosphodiester bond hydrolysis	13	2.05	5.32E-07
	meiotic chromosome segregation	16	2.52	1.84E-04
	mismatch repair	5	0.79	3.39E-04
**purple**	locomotion	148	17.03	5.05E-15
	nematode larval development	177	20.37	3.40E-11
	reproduction	191	21.98	3.47E-11
	embryo development ending in birth or egg hatching	244	28.08	2.50E-10
	small GTPase mediated signal transduction	18	2.07	1.69E-05
**violet**	G-protein coupled receptor signaling pathway	80	8.07	6.66E-18
	detection of chemical stimulus involved in sensory perception	46	4.64	7.00E-13
	sensory perception of chemical stimulus	32	3.23	3.63E-11
	detection of chemical stimulus involved in sensory perception of smell	34	3.43	6.91E-09
	olfactory behavior	35	3.53	1.05E-08

### Modulation of transcription factor expression in response to abeta accumulation

**Fig 7:**
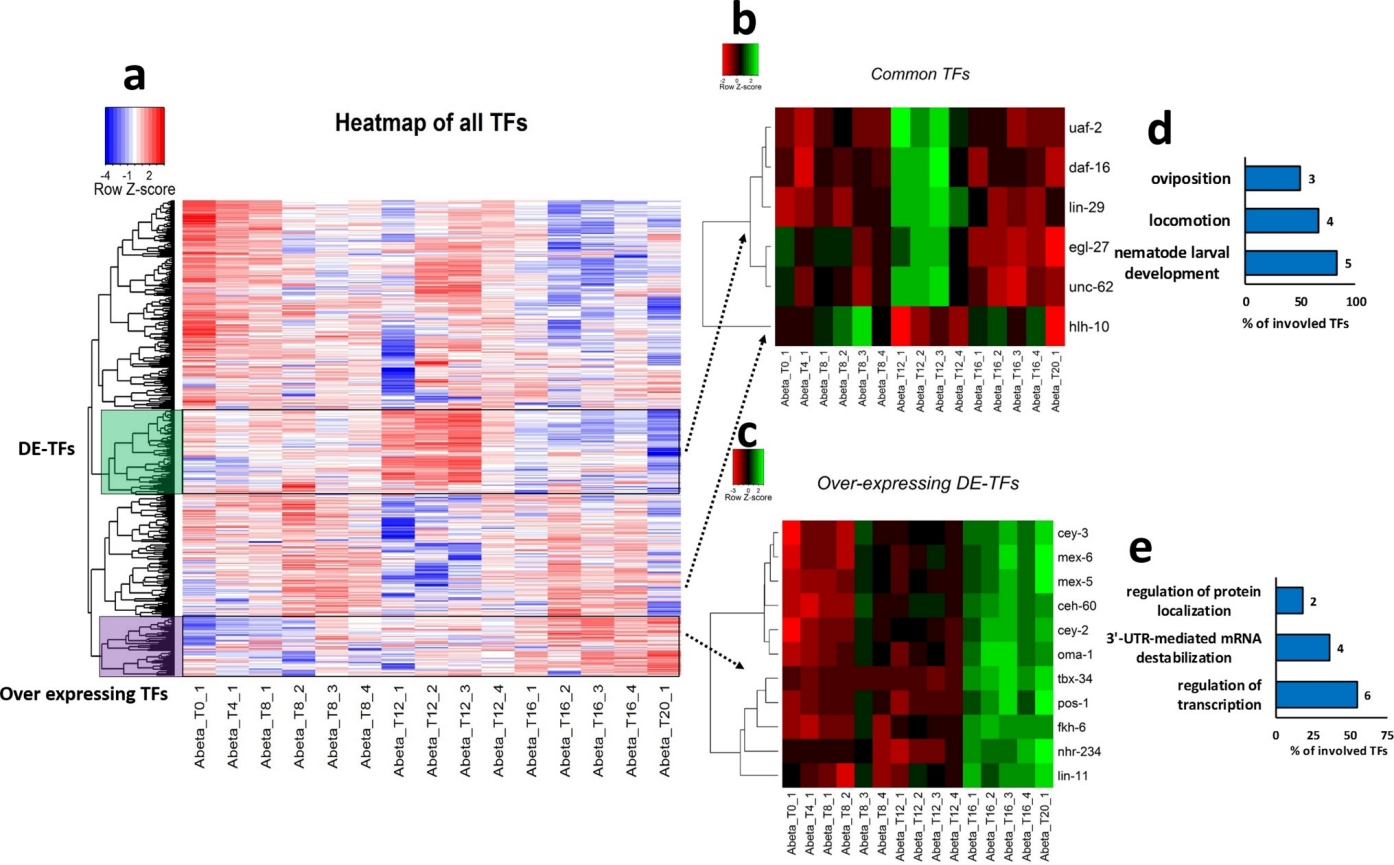
Clustering of TFs in transcriptome data obtained from Abeta expressing *C*. *elegans*. a) Heatmap showing clustering of all TFs based on similarity approach. We marked two clusters on the heatmap based on their expression pattern through successive stages. Either upregulation at T12 (these DE-TFs show over expression in at least three stages) or marked a group of TFs with low expression at the early time-points and over expressing in later stages. To highlight these differences, we have included b) The clustering of DE-TFs among at least three stages by correlation method. c) Clustering of up-regulated DEGs in T12 *vs*. T8 and T16 *vs*. T12 by correlation method. d and e) Show the top three biological processes (p-value ≤ 0.05) that DE-TFs and Over expressing TFs are involved in, respectively.

By comparing the 582 TFs proposed by [23] against the expression matrix prepared in the current study, 540 TFs were detected to be present in the transcriptome of *C*. *elegans* in response to Abeta accumulation at different stages. Cluster analysis based on similarity method has identified several clusters of TFs across the different time-points. However, only two of these clusters showed an altered pattern of expression according to the progression through time-points (Fig 7A). Many of the TFs were not differentially expressed, therefore we selected only the DE-TFs that are present at least in two comparisons. Six DE-TFs, including *uaf-2*, *daf-16*, *lin-29*, *egl-27*, *unc-62*, and *hlh-10* were detected and labeled as DE-TFs cluster. Interestingly the expression patterns for these DE-TFs followed similar trends in the comparisons, except for the *hlh-10* that showed slightly different expression trends to that of other DE-TFs (Fig 7B). In addition, to find common TFs that express similarly in response to accumulation of Abeta protein, a cluster was identified and labeled as over-expressing TFs, which contains only those DE-TFs that show accumulative trends through consecutive stages. These up-regulated DE-TFs were selected and re-clustered to identify those TFs that respond to the accumulation of Abeta protein. Over-expression was observed for the TFs, including *tbx-34*, *pos-1*, *oma-1*, *mex-6*, *ceh-60*, *mex-5*, *fkh-6*, *cey-2*, *nhr-234*, *lin-11*, and *cey-3* through successive time-points, therefore these TFs could be considered as the major regulators of gene regulation upon increases in the Abeta expression (Fig 7C).

### Specific responses to Abeta accumulation during development

**Fig 8:**
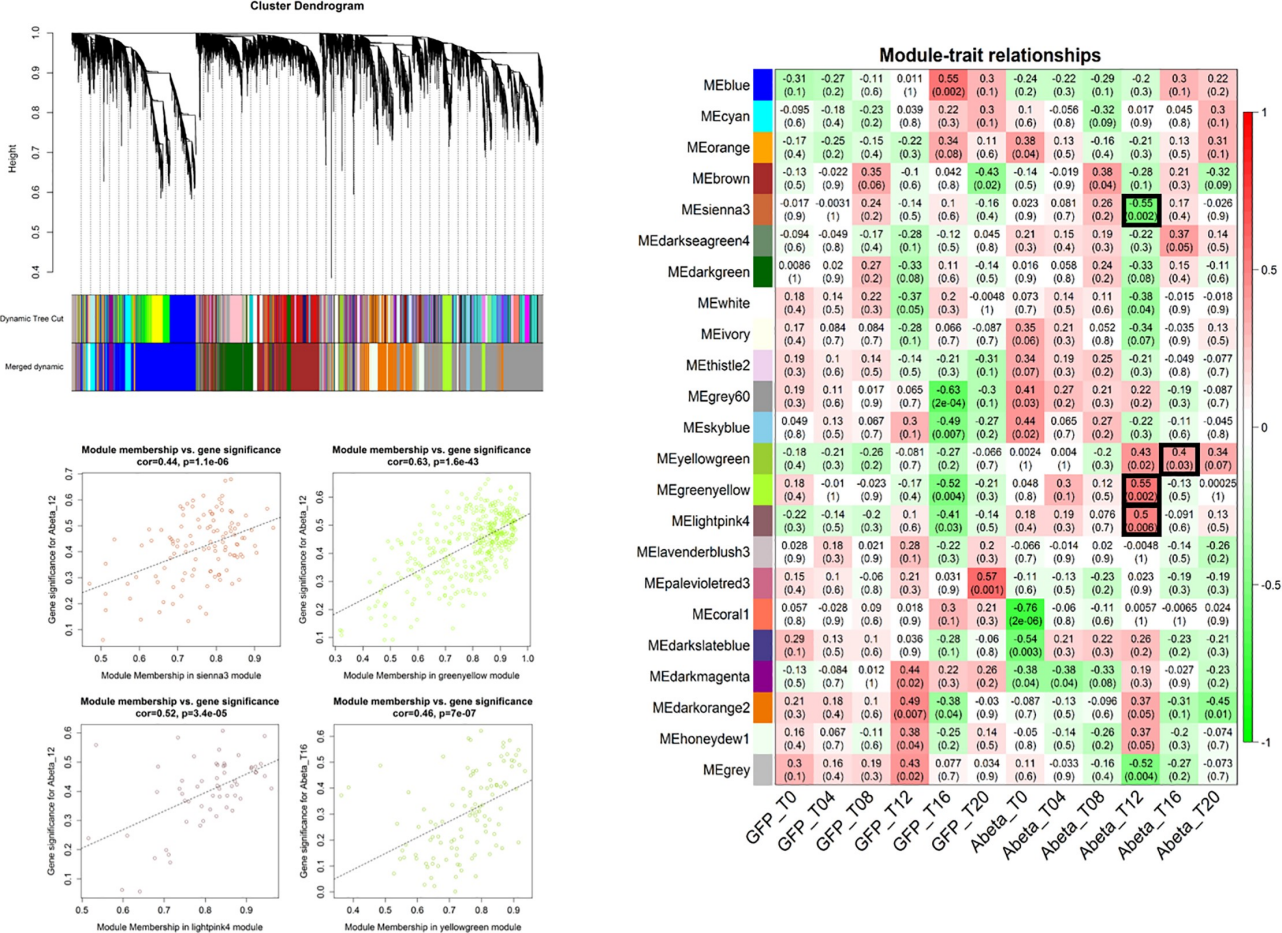
WGCNA analysis of the comparison between GFP and Abeta-GFP accumulation. There are clearly three modules related to Abeta accumulation. The GO of the members of these modules are presented in the S3 Fig Settings and thresholds are as Fig 5.

**Fig 9:**
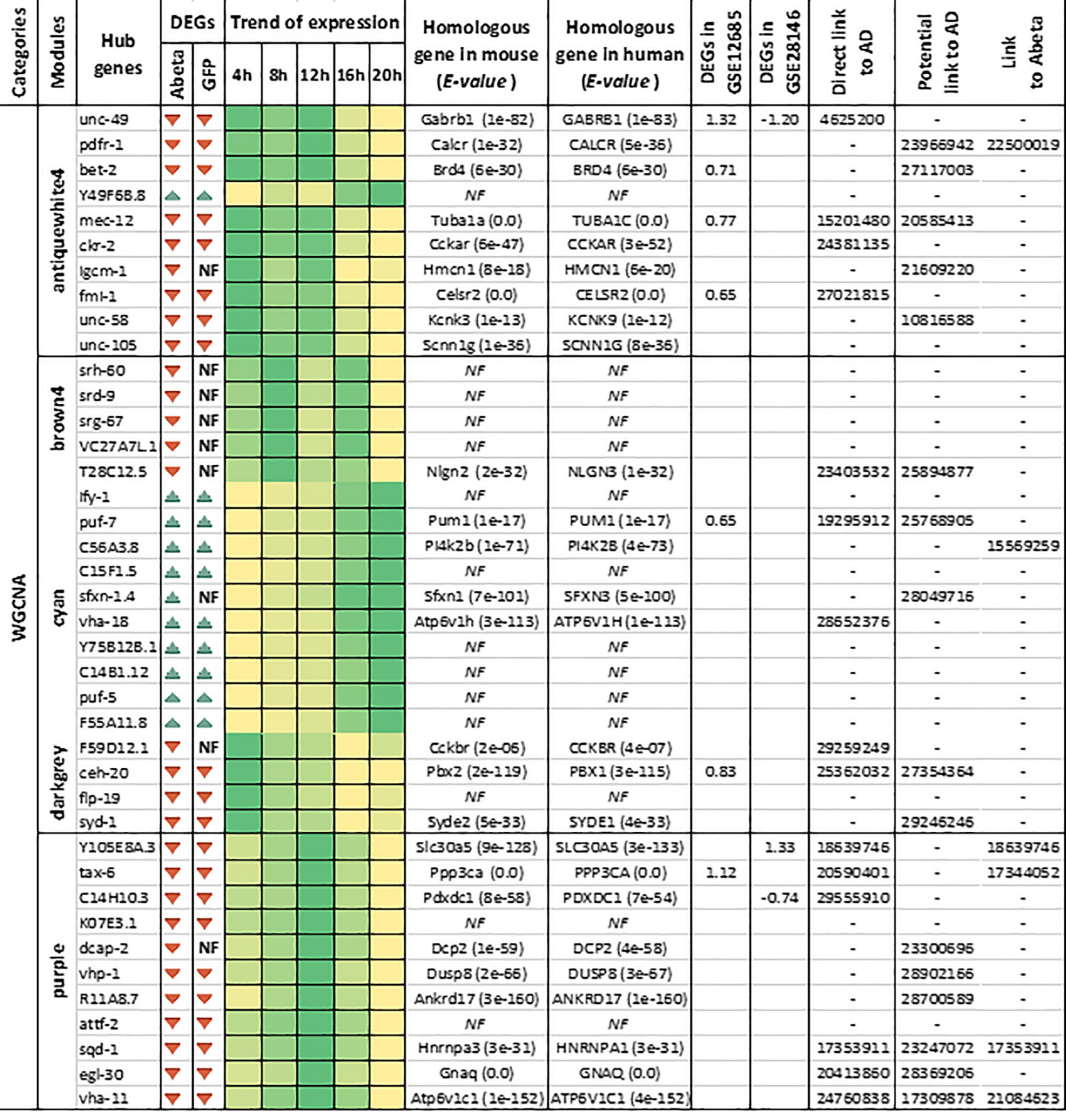
Hub genes from modules related to the Abeta expressing *C*. *elegans*. For each gene, expression trends through time-points (every 4 hours) and the mouse and human homologous genes were detected. For identified homologous genes, expression status in two datasets from human incipient Alzheimer’s disease, the direct relationship with Alzheimer’s disease, any potential role in Alzheimer’s or any neurodegenerative disease and Abeta presence were investigated. For each gene, higher intensity in color shows higher expression. *NF* indicates homologous genes are not found. Color bars indicate the trend of gene expression through time points.

**Fig 10:**
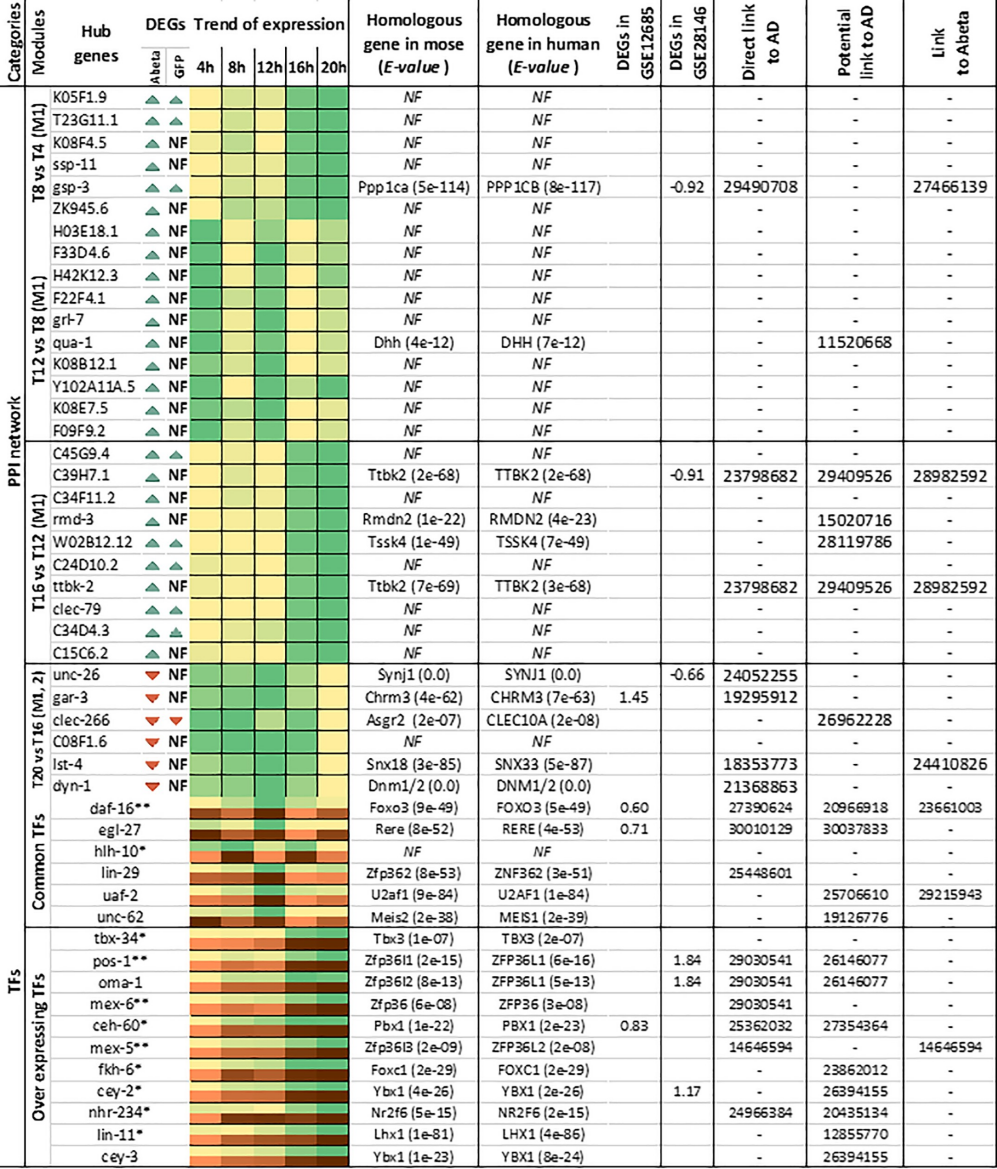
The top hub genes (Maximum 10) detected in the PPI networks at different stages of Abeta accumulation have been listed. For each gene, expression trends through time-points (every 4 hours), mouse and human homologous genes were extracted and presented. For identified homologous genes, expression status in two datasets from human incipient Alzheimer’s disease, the direct relationship with Alzheimer’s disease, any potential role in Alzheimer’s or association with Abeta accumulation were also included. For each gene, higher intensity in color shows higher expression. *NF* indicates homologous genes could not be found. * TFs responding to Abeta accumulation through development, ** TFs uniquely responding to Abeta accumulation when compared to GFP sample at the same developmental stage. Brown and green bars indicate trends of expression in response to GFP and Abeta accumulation, respectively. Darker color indicates more expression.

The time point comparisons we conducted in the previous sections do not rule out the possible impact of developmental stages on the observed perturbation of gene expression. To identify specific factors which solely respond to Abeta accumulation (and not developmental stages), we conducted similar analysis but instead of comparing each time point with the previous one, at each time-point we compared gene expression of Abeta-GFP expressing samples with those of GFP expressing counterpart (S1 and S2 Figs). Through WGCNA analysis we identified 3 modules specifically responding to Abeta accumulation and not GFP expression (Fig 8). The GO analysis of the members of these modules including “Sienna3”, “greenyellow” and “yellowgreen” are provided in S3 Fig Biological processes such as detection of chemicals, locomotion and metabolic process are among the most affected pathways. Interestingly, the specific responses to Abeta accumulation were evident at 12 and 16 hours after induction. We also extracted and analyzed the subnetworks of the involved modules (S4 Fig). The most important TFs and genes in the comparisons were compiled and presented in Figs 9 and 10, which also include literature review for the role of each entry.

### Homology analysis, gene relationships to AD and validation of results

Human and mouse orthologues for the hub genes and DE-TFs were identified through Blast analysis. Numerous worm genes had at least one orthologue in the human or mouse genome (Figs 9 and 10). Regarding WGCNA, only differentially expressed hub genes were selected and their orthologues were identified. Results showed that 65% (26 out of 40) of the hub genes detected by WGCNA in worm had an orthologue in either mouse or human or both organisms. Most orthologous genes were detected in modules labeled as “antiquewhite4” and “purple”. In contrast module “brown4” contained the lowest number of orthologues (Fig 9). Hub genes detected in the PPI networks of *C*. *elegans* had the lowest percentage of identified orthologues, where we could identify orthologues for only 11 out of 32 genes. However, when only DE-TFs were blasted against the human and mouse genomes, almost all of these TFs that affected by Abeta accumulation in worm had at least one orthologue in the other organisms, except for *hlh-10* (Fig 10).

In the next step, we reviewed the literature to assess if the orthologous genes, detected in human and mouse, are linked to AD. Additionally, expression patterns of orthologues were also investigated in two other independent datasets available for incipient AD. We found that 58% of the orthologues (15 out of 26) for the genes detected as a hub in the WGCNA analysis had a direct link to AD. In addition, we established indirect links to AD for the remainder of the orthologous genes in that analysis. Furthermore, 26% of the orthologous genes were directly linked to Abeta perturbation (Fig 9). Regarding the orthologous genes detected for the hub genes in the *C*. *elegans* PPI networks, 7 out of 11 had a direct link to AD, while, 6 out of 11 of these orthologous genes had a potential linkage to AD. Regarding the transcription factors, orthologues of DE-TFs were also associated with AD. We found that while gene expression perturbations for 56% of identified orthologues were reported in AD, 3 of these DE-TFs were directly linked to Abeta accumulation. Table 2 provides a convenient view of the hub genes, with information related to the function, cellular compartment, and the cell type or organ that the genes are expressed in, in addition to the function of their human orthologous genes. Interestingly, some of the genes are expressed in neural cells, however, they are also expressed in other tissues.

**Table 2:** Encoded protein, gene ontology and anatomical location of hub genes in worm and biological processes of their orthologues in human. All the Information are extracted from Uniprot (https://uniprot.org) and Wormbase (https://wormbase.org) databases. Anatomical location indicates cell/organ/regions that the genes are expressed in. Dash lines means no information has been found.

In worm					In human	
Gene symbol	Encoded protein	Cellular compartment	Function/process	Anatomical location	Human ortholog	Function/process
**gsp-3**	Serine/threonine-protein phosphatase PP1-gamma	Nuclear chromatin	Cell differentiation	Germ line/Sperm	*PPP1CB*	Cell cycle/Protein kinase binding
**qua-1**	Protein qua-1	Extracellular matrix	Cell-cell signaling	Excretory duct	*DHH*	Cell-cell signaling
**C39H7.1**	Uncharacterized protein	Nucleus	Peptidyl-serine phosphorylation	-	*TTBK2*	Cerebellum development
**rmd-3**	Regulator of Microtubule Dynamics	Nucleus	Regulator of Microtubule Dynamics	-	*RMDN2*	Regulator of microtubule dynamics
**W02B12.12**	Uncharacterized protein	Nucleus	Intracellular signal transduction/Kinase	-	*TSSK4*	Cell differentiation/Intracellular signal transduction
**ttbk-2**	Tau TuBulin Kinase	Nucleus/cytoplasm	Protein phosphorylation	-	*TTBK2*	Cerebellum development
**unc-26**	Synaptojanin	Cytoplasm	Neurotransmitter secretion	Neuron /Other regions	*SYNJ1*	Brain development/Neurotransmitter transport
**gar-3**	Muscarinic acetylcholine receptor gar-3	Membrane	Action potential	Neuron	*CHRM3*	Cell population proliferation/Chemical synaptic transmission
**clec-266**	C-type LECtin	Membrane	Carbohydrate binding	Intestinal cell	*CLEC10A*	Immune response
**lst-4**	Sorting nexin lst-4	Cytoplasm	Mitotic cell cycle/Apoptosis	Embryonic cell/Gonad	*SNX33*	Endocytosis/Cleavage furrow formation
**dyn-1**	Dynamin	Cytoplasm	Endocytosis	Neuron/ Intestine	*DNM1/2*	Endocytosis
**daf-16**	Forkhead box protein O	Nucleus	Aging	Neuron/Vulva	*FOXO3*	Aging/Cellular response to amyloid-beta
**egl-27**	Egg-laying defective protein 27	Nucleus	Cell fate specification	Neuron /Other regions	*RERE*	Branching morphogenesis of a nerve
**lin-29**	Transcription factor	Nucleus	Regulation of development/Apoptosis	Anchor cell/B cell	*ZNF362*	Transcription regulation
**uaf-2**	U2AF splicing factor	Spliceosomal complex	mRNA splicing	Neuron /Other regions	*U2AF1*	mRNA 3'-end processing
**unc-62**	Homeobox protein unc-62	Nucleus	Mesodermal cell fate specification	Neuron/other regions	*MEIS1*	Angiogenesis/Locomotory behavior
**pos-1**	Cytoplasmic zinc-finger protein	Cytoplasm	Cell fate specification	Neuron/Other regions	*ZFP36L1*	3'-UTR-mediated mRNA destabilization/Apoptotic process
**oma-1**	CCCH-type zinc finger protein oma-1	Nucleus/cytoplasm	Regulation of translation/Oocyte growth	Neuron/Other regions	*ZFP36L1*	3'-UTR-mediated mRNA destabilization/Apoptotic process
**mex-6**	Zinc finger protein mex-6	Cytoplasm	Regulation of protein localization	Germ line	*ZFP36*	3'-UTR-mediated mRNA destabilization
**ceh-60**	C. Elegans Homeobox	Nucleus	Regulation of transcription	Amphid neuron	*PBX1*	Transcription regulation/ Differentiation
**mex-5**	Zinc finger protein mex-5	Cytoplasm	Regulation of protein localization	Neuron/Other regions	*ZFP36L2*	3'-UTR-mediated mRNA destabilization
**fkh-6**	ForKHead transcription factor family	Nucleus	Regulation of transcription	Gonad	*FOXC1*	Anatomical structure morphogenesis
**cey-2**	C. Elegans Y-box	Ribonucleoprotein complex	Nucleic acid binding	Germ line/Pharynx	*YBX1*	CRD-mediated mRNA stabilization
**nhr-234**	Nuclear Hormone Receptor family	Nucleus	Regulation of transcription	-	*NR2F6*	Neuron development/Signal transduction
**lin-11**	Protein lin-11	Nucleus	Axonal fasciculation	Neuron	*LHX1*	Anatomical structure morphogenesis/Motor neuron axon guidance
**cey-3**	C. Elegans Y-box	Ribonucleoprotein complex	-	Neuron/Other regions	*YBX1*	CRD-mediated mRNA stabilization
**unc-49**	Ionotropic GABA receptor subunit UNC-49C	Membrane/synapse	Chemical synaptic transmission	Neuron/Muscle	*GABRB1*	Nervous system neuron development/Chemical synaptic transmission
**pdfr-1**	Calcitonin receptor-like protein 1	Membrane	Signal transduction	Neuron/Muscle	*CALCR*	Amylin receptor signaling pathway
**bet-2**	BET (Two bromodomains) family protein	-	-	-	*BRD4*	Cellular response to DNA damage stimulus/Chromatin organization
**mec-12**	Tubulin alpha-3 chain	Microtubule/axon	Microtubule cytoskeleton organization	Neuron/Intestine	*TUBA1C*	Cytoskeleton-dependent intracellular transport
**ckr-2**	CholecystoKinin Receptor homolog	Membrane	Neuropeptide signaling pathway	Neuron	*CCKAR*	Axonogenesis
**igcm-1**	ImmunoGlobulin-like Cell adhesion Molecule family	Membrane	-	Neuron/Other regions	*HMCN1*	Cell cycle/Cell division
**fmi-1**	FlaMIngo (Cadherin plus 7TM domain) homolog	Membrane/axon	G protein-coupled receptor signaling pathway/Axon guidance	Neuron	*CELSR2*	Dendrite morphogenesis
**unc-58**	Uncoordinated protein 58	Membrane	Ion transport/Muscle contraction	Neuron	*KCNK9*	Potassium ion transport/Stabilization of membrane potential
**T28C12.5**	Uncharacterized protein	-	Express in neuron	Neuron	*NLGN3*	Axon extension/Adult behavior
**puf-7**	Pumilio domain-containing protein 7	-	Regulation of translation/Cell cycle	-	*PUM1*	Adult locomotory behavior/mRNA destabilization
**sfxn-1.4**	Sideroflexin	Mitochondrion membrane	Ion transport	-	*SFXN3*	Iron ion homeostasis
**vha-18**	Probable V-type proton ATPase subunit H 1	Cytoplasm	Ion transport	-	*ATP6V1H*	Endocytosis/Ion transmembrane transport
**F59D12.1**	Uncharacterized protein	Membrane	G protein-coupled receptor signaling pathway	-	*CCKBR*	Cell population proliferation/Cholecystokinin signaling pathway
**ceh-20**	Homeobox protein ceh-20	Nucleus	DNA binding/Mesoderm development	Neuron/Other regions	*PBX1*	Transcription regulation/ Differentiation
**syd-1**	Rho GTPase-activating protein syd-1	Cell junction/Synaptic	Signal transduction/Axo-dendritic transport	Neuron	*SYDE1*	Activation of gtpase activity/Regulation of cytoskeleton organization
**Y105E8A.3**	Uncharacterized protein	Endoplasmic reticulum	Transmembrane transport	-	*SLC30A5*	Ion transport
**tax-6**	Serine/threonine-protein phosphatase 2B catalytic subunit	Nucleus/cytoplasm	Calcineurin-mediated signaling/Chemosensory behavior	Neuron/Other regions	*PPP3CA*	Brain development/Calcium ion transport
**C14H10.3**	Uncharacterized protein	Endoplasmic reticulum	Ameboidal-type cell migration	-	*PDXDC1*	Ameboidal-type cell migration/Sphingolipid metabolism
**dcap-2**	mRNA-decapping enzyme 2	Cytoplasm	Reproduction	-	*DCP2*	mRNA catabolic process
**vhp-1**	Tyrosine-protein phosphatase vhp-1	Nucleus/cytoplasm	Control of MAPK activity/Axon regeneration	Neuron/Other regions	*DUSP8*	Inactivation of MAPK activity
**R11A8.7**	Ankyrin repeat and KH domain-containing protein mask-1	Cytoplasm	RNA binding	-	*ANKRD17*	Innate immune response/RNA binding
**sqd-1**	Homologous to Drosophila SQD (Squid) protein	Ribonucleoprotein complex	RNA binding	-	*HNRNPA1*	Cellular response to glucose starvation/mRNA splicing
**egl-30**	EGL-30	Cytoplasm	Nematode larval development/Activation of immune response	Neuron/Muscle	*GNAQ*	Action potential/Entrainment of circadian clock
**vha-11**	V-type proton ATPase subunit C	Cytoplasm	Ion transport/Multicellular organism development	Intestine/Hypodermis	*ATP6V1C1*	Insulin receptor signaling pathway ion transmembrane transport

**Fig 11:**
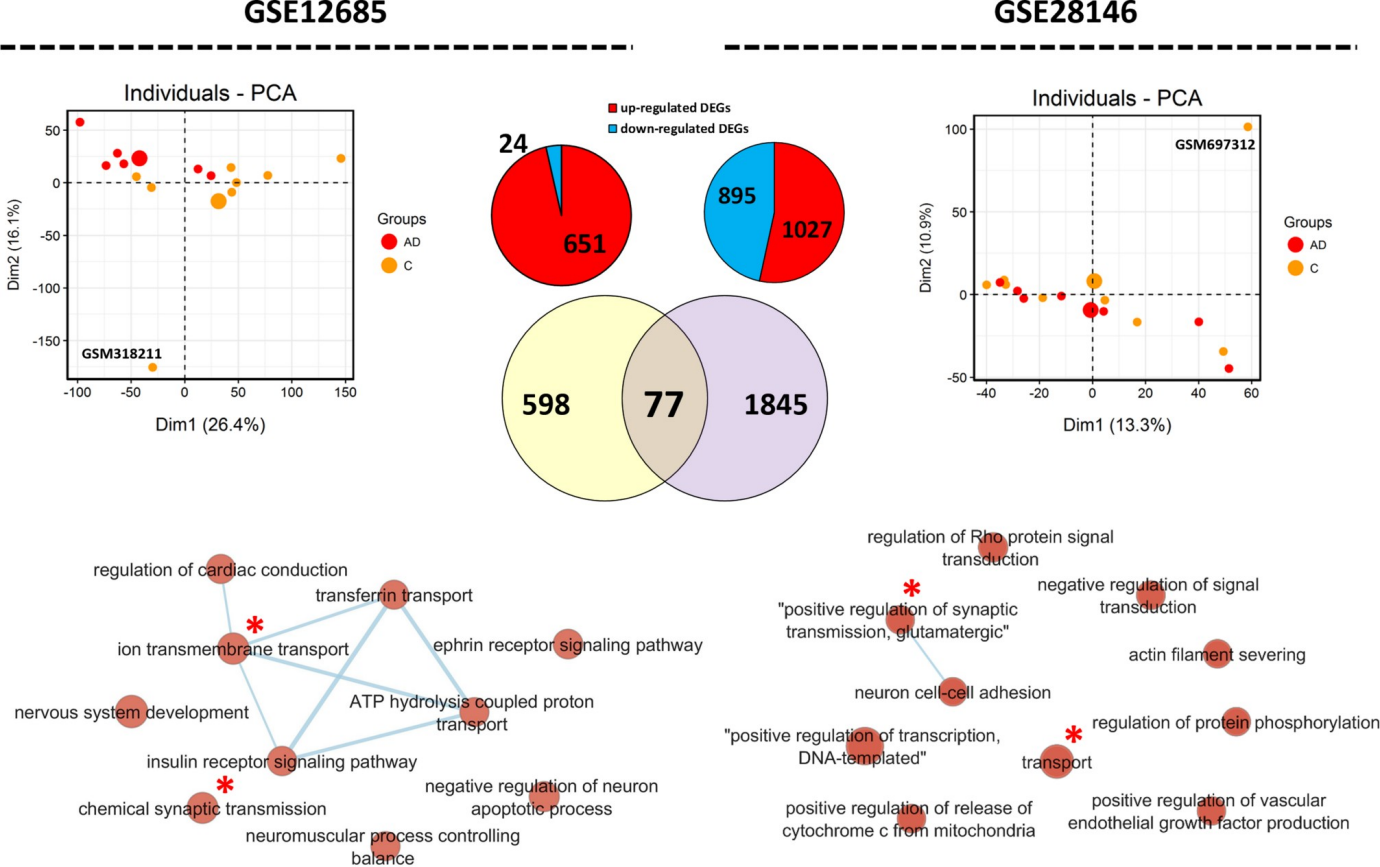
Transcriptome analysis of samples obtained from human data at incipient Alzheimer’s disease. We have analyzed two datasets, GSE12685 and GSE28146. PCA was performed, which as expected showed noticeable heterogeneity among samples. DEGs were detected using GEO2R tool. 77 common DEGs were detected between the two datasets. Enrichment map of biological processes show that transport (transmembrane) and actin organization are common in both datasets (indicate by red stars). Nodes in larger size contain a larger number of involved DEGs in the biological process. In the PCA graph, large dots are average of all the samples of that group.

We validated our analysis with datasets related to incipient AD. After removing outlier arrays, as totally separated samples from the clusters (GSM318211, and GSM697312 from GSE12685 and GSE28146, respectively), DEGs, common DEGs, and biological process were detected (Fig 11). Analyzing these datasets showed variation in the transcriptome profile between different studies in the incipient stage of AD. Processes related to transport and synaptic transmission were common in both datasets. Comparing the list of orthologous genes with the list of genes obtained from these two datasets identified that several of human orthologues of the Abeta-responding genes in the worm undergo gene expression modifications in these datasets, particularly GSE12685. However, when we analyzed the trends of gene expression, different trends of expression were observed (Figs 9 and 10). Most orthologous genes obtained from WGCNA and DE-TFs analysis of worm data were found to be differentially expressed in the human AD datasets (Fig 10).

## Discussion

Owing to the high prevalence and mortality rate, AD has been the center of attention for many neuroscience researchers. Although numerous genes, proteins, and molecular processes related to the pathogenicity of AD have been identified, the key genes and proteins involved in the onset of the disease have remained mostly elusive [1, 27]. The main obstacle in this type of research is inaccessibility of brain tissue samples at the early stages of AD, which is the asymptomatic period of the disease. As an alternative approach, early molecular alterations in AD can be examined in animal models that develop disease symptoms. Among these animal models, *C*. *elegans* expressing human Abeta protein enables detection of changes in the expression of genes in response to Abeta aggregation. In spite of the genome similarities between human and worm, there is a large evolutionary and structurally distance between these animals that brings into question the reliability of associations between the genes obtained from worm to that of human disease.

In order to address this issue, we used two network approaches, WGCNA and PPI, to detect important genes and pathways that respond to the accumulation of Abeta in *C*. *elegans*. PPI networks were constructed using DEGs from analysis at different temporal stages of Abeta-GFP expression compared to GFP-expressing control samples. Gene modules and hub nodes related to each stage were obtained from WGCNA, which uses original expression values rather than differentially expressed values. Generally, we found several biological processes relevant to AD in both analyses of WGCNA or PPI. Transcriptome alterations in response to Abeta accumulation were most evident at the later time-points (T12 and T16). However, the alteration of gene expression decreases from T12 onwards, suggesting that Abeta protein accumulation might cause gradual alterations to gene expression and exerts its impacts rather early in the progression of AD. This observation could explain why the onset of AD could happen many years before recognizing symptoms [1].

We could link many of the identified biological processes in the *C*. *elegans* response to Abeta protein directly to AD in human, including metabolic processes [28], protein phosphorylation [29], G-protein coupled receptors [30], meiotic chromosome segregation and cell cycle [31], and regulation of transcription [32]. These similarities reflect possible evolutionary conservation in the molecular mechanisms of responses to Abeta in both worms and humans. There were also alterations in other processes including the molting cycle, collagen, and cuticulin-based cuticle, olfactory behavior, body morphogenesis, locomotion, hermaphrodite genitalia development, and nematode larval development. Additionally, similar to what the original study reported regarding detection of heat shock proteins in response to Abeta, we detected genes responsible for heat shock responses at stage 2 (T12 *vs*. T08) (Figs 2 and 3) [11]. Interestingly, similar to what we found here, a previous study indicated that overexpression of APP-related protein (APL-1) in neurons disrupts olfactory behaviors in *C*. *elegans* [33].

*C*. *elegans* is different from humans in many anatomical, molecular and physiological characteristics [34]. Most notably, *C*. *elegans* lacks β-secretase or Abeta expressing genes in its genome [10]. Additionally, in these two organisms, Abeta aggregation occurs in different tissues; in muscles of *C*. *elegans* model (in this study CL4176) compared to the brain in human [11]. In spite of these differences, our systems-level observations indicate that Abeta aggregation affect somewhat similar processes in both systems.

Responses to Abeta aggregation are expected to be exerted via hub genes in networks constructed for these systems. Three modules (“antiquewhite4”, “cyan”, and “purple”), identified by WGCNA, contained the most affected, differentially expressed hub genes (Fig 6). We observed synchronized trends of expression among these hub genes as well as hub proteins that were identified in the PPI network analysis. Next, the relationship between these hub genes and proteins to AD or Abeta was investigated by mining previously published studies. Although there are publications describing molecular mechanisms of *C*. *elegans* models for AD [35, 36], as we expected, there are few publications studying AD or Abeta accumulation in *C*. *elegans*. We only found two hub genes in *C*. *elegans* that have already been previously reported to be associated to Abeta accumulation, including *mec-12* that encodes alpha tubulin subunit [37], and *daf-16* that mediates proteostasis to tolerate β-amyloid toxicity [38]. Therefore, for the first time, we have established some degree of association between these hub genes and proteins with Abeta accumulation in *C*. *elegans*.

Following Abeta accumulation, the expression level of multiple TFs may be affected, and as a result gene regulatory networks in cells would be perturbed. We found that several transcription factors are deregulated in response to Abeta accumulation (Fig 7). This finding is in agreement with a previous study on the role of *daf-16* in response to Abeta aggregation in *C*. *elegans* [38]. Additionally, *daf-16* and its orthologous FOXO genes are major regulators of aging and stress responses [39, 40]. It has been shown that mutation in *daf-16* increases dopaminergic neurodegeneration, however, DAF-16 is not the only neuroprotective agent [41]. In our study, *daf-16* was not continuously over-expressed and the highest expression occurred at T12 which could be in response to severe muscle paralysis. The importance of two other TFs, EGL-27 and NUC-62, in postponing aging and increase in *C*. *elegans* lifespan have also been reported [42, 43]. The important roles of these TFs in the aging process and their alteration by Abeta aggregation, suggest potentially crucial roles for them and their human orthologous TFs in AD. Interestingly, the literature review for the human orthologous of the over-expressing TFs showed a high percentage of association with AD (Fig 10). RERE is orthologue of EGL-27 that is involved in AD has been detected by bioinformatical methods and gene expression analysis [44]. ZNF362 and PBX1 are human orthologue of LIN-29 and CEH-60 that are also deregulated in post-mortem AD patient brain samples [45, 46]. Deletion of Nr2f6 (Ear2), the mouse orthologous of NHR-234, leads to defects in early memory and learning in the mouse model for AD, APP/PS1 [47]. This point suggests that these over-expressing TFs are important in responding to Abeta aggregation and start their activity later than other TFs.

By searching for orthologues in human or mouse genome we found that many of the hub genes have orthologous counterparts in these organisms that are directly or potentially involved in AD (References are included in Figs 9 and 10). Generally, in WGCNA, the hub genes in modules related to transport and determination of lifespan (“antiquwhite4”) and nematode larval development “purple” had the highest number of orthologs in the human and mouse genome. Interestingly, gene ontology of the orthologous genes revealed that they are present in pathways including neuroactive ligand-receptor interaction plus glutamatergic and dopaminergic synapses. Among these genes, we found some orthologues with a remarkable role in AD. For example, *TTBK2* (the *C*. *elegans C39H7*.*1* orthologue) over-expression reduces phosphorylation of tau protein in AD [48]. While *CCKAR* (*ckr-2* in *C*. *elegans*) has been identified as a biomarker of AD [49]. Another important gene, *HNRNPA1* the human orthologue for *sqd-1*, is involved in alternative splicing of APP genes that could be targeted for reducing senile plaque formation [50]. Polymorphism in the *PPP3CA* gene (orthologue of *tax-6 in* in *C*. *elegans*) has been reported in AD patients, indicating a role for this gene in the disease [51]. APP endocytosis and APP alpha-secretase cleavage is under *SNX33* regulation, which is an endocytic protein orthologue of *lst-4* in worms [52]. Treusch and colleagues studied toxicity of Abeta in yeast and found several genes that modify Abeta toxicity. Interestingly, they found orthologues of these genes are modifiers of Abeta toxicity in glutamatergic neurons of *C*. *elegans* and primary rat cortical neurons [53]. Two of our hub genes, *unc-26* and *lst-4*, are present in this list. Remarkably, their orthologues in mammalians are also linked to AD [54, 55]. Out of the genes discussed above with a potential role in AD, *C39H7*.*1*, *lst-4*, and *daf-16* appear to specifically respond directly to the accumulation of Abeta (Figs 9 and 10), while others could be affected either by Abeta or developmental progression. However, we could not rule out a combinational impact of both these factors on gene expression changes. On the other hand, *pos-1*, *nhr-234*, mex*-5* and mex-6 are all overexpressed in response to Abeta accumulation and all have been directly or indirectly linked to AD [47, 56–59]. This shows the efficiency of our approach to detect the most important factors in response to Abeta accumulation.

It has been shown that gene regulatory networks are conserved across metazoa [60]. As expected, altered TFs in *C*. *elegans* had orthologues in human. Interestingly, many of the human orthologous TFs are directly associated with AD or other neurodegenerative disorders. This suggests that conserved regulatory networks are involved in responses to Abeta aggregation and AD pathogenesis. We reasoned that deregulation of the human orthologous genes might be altered during early AD. To examine this, we have selected two datasets from incipient AD in human. We found deregulation in a number of these genes, however, opposite trends of expression were observed in different datasets. Unexpectedly, we found that two datasets are also different in DEGs and expression patterns (Fig 11). Many of these genes are previously reported, indicating the involvement of these homologous genes in responses to Abeta accumulation in both human and worm (PMID of the references are included in Figs 9 and 10).

In conclusion, using systems biology we have identified important genes and biological processes in *C*. *elegans* that respond to Abeta aggregation. These results could be useful for examining the molecular mechanisms involved in Abeta aggregation as a main cause of AD, which may be applied as further diagnostic or therapeutic targets. Additionally, we suggest *C*. *elegans* could be considered as a useful model for studying early molecular events in AD because of the evolutionary relationships to AD in humans documented in this study.

## Supporting information

S1 Fig Venn diagram showing the uniquely and shared DEG between GFP and Abeat expressing worms when compared to the control at each time point. (TIF)

S2 Fig Bar graph showing the up and down regulation of DEG for the GFP and Abeta expressing worms at each time point. (TIF)

S3 Fig Biological processes of Abeta responsive modules. Three unique modules were detected for Abeta responsive gene list (Fig 8) were subjected to GO analysis.
(TIF)

S4 Fig Subnetworks for the detected modules in response to Abeta expression. The analysis was included in Figs 9 and 10 in the main text.
(TIF)

## References

[1] Murphy MP, LeVine H III. Alzheimer's disease and the amyloid-β peptide. Journal of Alzheimer's Disease. 2010;19(1):311–23. 10.3233/JAD-2010-1221 20061647 PMC2813509

[2] Zhang X, Li Y, Xu H, Zhang Y-w. The γ-secretase complex: from structure to function. Frontiers in cellular neuroscience. 2014;8:427 10.3389/fncel.2014.00427 25565961 PMC4263104

[3] Iwatsubo T, Odaka A, Suzuki N, Mizusawa H, Nukina N, Ihara Y. Visualization of Aβ42 (43) and Aβ40 in senile plaques with end-specific Aβ monoclonals: evidence that an initially deposited species is Aβ42 (43). Neuron. 1994;13(1):45–53. 8043280 10.1016/0896-6273(94)90458-8

[4] Baruch-Suchodolsky R, Fischer B. Aβ40, either soluble or aggregated, is a remarkably potent antioxidant in cell-free oxidative systems. Biochemistry. 2009;48(20):4354–70. 10.1021/bi802361k 19320465

[5] Pearson HA, Peers C. Physiological roles for amyloid β peptides. The Journal of physiology. 2006;575(1):5–10. 16809372 10.1113/jphysiol.2006.111203 PMC1819417

[6] Tabaton M, Zhu X, Perry G, Smith MA, Giliberto L. Signaling effect of amyloid-β42 on the processing of AβPP. Experimental neurology. 2010;221(1):18–25. 10.1016/j.expneurol.2009.09.002 19747481 PMC2812589

[7] Do Carmo S, Cuello AC. Modeling Alzheimer’s disease in transgenic rats. Molecular neurodegeneration. 2013;8(1):37. 24161192 10.1186/1750-1326-8-37 PMC4231465

[8] Hall AM, Roberson ED. Mouse models of Alzheimer's disease. Brain research bulletin. 2012;88(1):3–12. 10.1016/j.brainresbull.2011.11.017 22142973 PMC3546481

[9] Prüßing K, Voigt A, Schulz JB. Drosophila melanogaster as a model organism for Alzheimer’s disease. Molecular neurodegeneration. 2013;8(1):35. 24267573 10.1186/1750-1326-8-35 PMC4222597

[10] Link CD. C. elegans models of age-associated neurodegenerative diseases: lessons from transgenic worm models of Alzheimer’s disease. Experimental gerontology. 2006;41(10):1007–13. 10.1016/j.exger.2006.06.059 16930903

[11] Hassan WM, Dostal V, Huemann BN, Yerg JE, Link CD. Identifying Aβ-specific pathogenic mechanisms using a nematode model of Alzheimer's disease. Neurobiology of aging. 2015;36(2):857–66. 10.1016/j.neurobiolaging.2014.10.016 25457027 PMC4315719

[12] Parikshak NN, Gandal MJ, Geschwind DH. Systems biology and gene networks in neurodevelopmental and neurodegenerative disorders. Nature Reviews Genetics. 2015;16(8):441 10.1038/nrg3934 26149713 PMC4699316

[13] van Dam S, Võsa U, van der Graaf A, Franke L, de Magalhães JP. Gene co-expression analysis for functional classification and gene–disease predictions. Briefings in bioinformatics. 2017. 10.1093/bib/bbw139 PMC6054162 28077403

[14] Langfelder P, Horvath S. WGCNA: an R package for weighted correlation network analysis. BMC bioinformatics. 2008;9(1):559. 19114008 10.1186/1471-2105-9-559 PMC2631488

[15] Sevimoglu T, Arga KY. The role of protein interaction networks in systems biomedicine. Computational and structural biotechnology journal. 2014;11(18):22–7. 10.1016/j.csbj.2014.08.008 25379140 PMC4212283

[16] Kiddle SJ, Sattlecker M, Proitsi P, Simmons A, Westman E, Bazenet C, et al Candidate blood proteome markers of Alzheimer's disease onset and progression: a systematic review and replication study. Journal of Alzheimer's Disease. 2014;38(3):515–31. 10.3233/JAD-130380 24121966

[17] Williams C, Shai RM, Wu Y, Hsu Y-H, Sitzer T, Spann B, et al Transcriptome analysis of synaptoneurosomes identifies neuroplasticity genes overexpressed in incipient Alzheimer's disease. PloS one. 2009;4(3):e4936 10.1371/journal.pone.0004936 19295912 PMC2654156

[18] Blalock EM, Buechel HM, Popovic J, Geddes JW, Landfield PW. Microarray analyses of laser-captured hippocampus reveal distinct gray and white matter signatures associated with incipient Alzheimer's disease. Journal of chemical neuroanatomy. 2011;42(2):118–26. 10.1016/j.jchemneu.2011.06.007 21756998 PMC3163806

[19] Shannon P, Markiel A, Ozier O, Baliga NS, Wang JT, Ramage D, et al Cytoscape: a software environment for integrated models of biomolecular interaction networks. Genome research. 2003;13(11):2498–504. 10.1101/gr.1239303 14597658 PMC403769

[20] Bastian M, Heymann S, Jacomy M. Gephi: an open source software for exploring and manipulating networks. Icwsm. 2009;8(2009):361–2.

[21] Szklarczyk D, Morris JH, Cook H, Kuhn M, Wyder S, Simonovic M, et al The STRING database in 2017: quality-controlled protein–protein association networks, made broadly accessible. Nucleic acids research. 2016:gkw937. 10.1093/nar/gkw937 PMC5210637 27924014

[22] Nepusz T, Yu H, Paccanaro A. Detecting overlapping protein complexes in protein-protein interaction networks. Nature methods. 2012;9(5):471 10.1038/nmeth.1938 22426491 PMC3543700

[23] Reece-Hoyes JS, Deplancke B, Shingles J, Grove CA, Hope IA, Walhout AJ. A compendium of Caenorhabditis elegans regulatory transcription factors: a resource for mapping transcription regulatory networks. Genome biology. 2005;6(13):R110 10.1186/gb-2005-6-13-r110 16420670 PMC1414109

[24] Huang DW, Sherman BT, Lempicki RA. Systematic and integrative analysis of large gene lists using DAVID bioinformatics resources. Nature protocols. 2008;4(1):44. 10.1038/nprot.2008.211 19131956

[25] Merico D, Isserlin R, Stueker O, Emili A, Bader GD. Enrichment map: a network-based method for gene-set enrichment visualization and interpretation. PloS one. 2010;5(11):e13984 10.1371/journal.pone.0013984 21085593 PMC2981572

[26] Link CD. C. elegans models of age-associated neurodegenerative diseases: lessons from transgenic worm models of Alzheimer's disease. Experimental gerontology. 2006;41(10):1007–13. Epub 2006/08/26. 10.1016/j.exger.2006.06.059 . 16930903

[27] Hersi M, Irvine B, Gupta P, Gomes J, Birkett N, Krewski D. Risk factors associated with the onset and progression of Alzheimer’s disease: a systematic review of the evidence. Neurotoxicology. 2017;61:143–87. 10.1016/j.neuro.2017.03.006 28363508

[28] Cai H, Cong W-n, Ji S, Rothman S, Maudsley S, Martin B. Metabolic dysfunction in Alzheimer's disease and related neurodegenerative disorders. Current Alzheimer Research. 2012;9(1):5–17. 22329649 10.2174/156720512799015064 PMC4097094

[29] Oliveira J, Costa M, de Almeida MSC, da Cruz e Silva OA, Henriques AG. Protein Phosphorylation is a Key Mechanism in Alzheimer’s Disease. Journal of Alzheimer's Disease. 2017;58(4):953–78. 10.3233/JAD-170176 28527217

[30] Thathiah A, De Strooper B. The role of G protein-coupled receptors in the pathology of Alzheimer's disease. Nature Reviews Neuroscience. 2011;12(2):73 10.1038/nrn2977 21248787

[31] Potter H. Cell cycle and chromosome segregation defects in Alzheimer’s disease Cell-Cycle Mechanisms and Neuronal Cell Death: Springer; 2005 p. 55–78.

[32] Chen X-F, Zhang Y-w, Xu H, Bu G. Transcriptional regulation and its misregulation in Alzheimer’s disease. Molecular brain. 2013;6(1):44. 24144318 10.1186/1756-6606-6-44 PMC3854070

[33] Ewald CY, Cheng R, Tolen L, Shah V, Gillani A, Nasrin A, et al Pan-neuronal expression of APL-1, an APP-related protein, disrupts olfactory, gustatory, and touch plasticity in Caenorhabditis elegans. Journal of Neuroscience. 2012;32(30):10156–69. 10.1523/JNEUROSCI.0495-12.2012 22836251 PMC3698849

[34] Shen P, Yue Y, Zheng J, Park Y. Caenorhabditis elegans: A Convenient In Vivo Model for Assessing the Impact of Food Bioactive Compounds on Obesity, Aging, and Alzheimer's Disease. Annual review of food science and technology. 2018;9:1–22. 10.1146/annurev-food-030117-012709 29261338

[35] Ewald CY, Li C. Caenorhabditis elegans as a model organism to study APP function. Experimental brain research. 2012;217(3–4):397–411. 10.1007/s00221-011-2905-7 22038715 PMC3746071

[36] Niwa R, Zhou F, Li C, Slack FJ. The expression of the Alzheimer's amyloid precursor protein-like gene is regulated by developmental timing microRNAs and their targets in Caenorhabditis elegans. Developmental biology. 2008;315(2):418–25. 10.1016/j.ydbio.2007.12.044 18262516 PMC2307910

[37] Ewald CY, Li C. Understanding the molecular basis of Alzheimer’s disease using a Caenorhabditis elegans model system. Brain Structure and Function. 2010;214(2–3):263–83. 10.1007/s00429-009-0235-3 20012092 PMC3902020

[38] Wang X, Cao M, Dong Y. Royal jelly promotes DAF-16-mediated proteostasis to tolerate β-amyloid toxicity in C. elegans model of Alzheimer's disease. Oncotarget. 2016;7(34):54183 10.18632/oncotarget.10857 27472466 PMC5342333

[39] Sun X, Chen W-D, Wang Y-D. DAF-16/FOXO transcription factor in aging and longevity. Frontiers in pharmacology. 2017;8:548 10.3389/fphar.2017.00548 28878670 PMC5572328

[40] Hesp K, Smant G, Kammenga JE. Caenorhabditis elegans DAF-16/FOXO transcription factor and its mammalian homologs associate with age-related disease. Experimental gerontology. 2015;72:1–7. 10.1016/j.exger.2015.09.006 26363351

[41] Knight AL, Yan X, Hamamichi S, Ajjuri RR, Mazzulli JR, Zhang MW, et al The glycolytic enzyme, GPI, is a functionally conserved modifier of dopaminergic neurodegeneration in Parkinson's models. Cell metabolism. 2014;20(1):145–57. Epub 2014/06/03. 10.1016/j.cmet.2014.04.017 24882066 PMC4097176

[42] Xu X, Kim SK. The GATA transcription factor egl-27 delays aging by promoting stress resistance in Caenorhabditis elegans. PLoS genetics. 2012;8(12):e1003108 10.1371/journal.pgen.1003108 23271974 PMC3521710

[43] Sagi D. The addition of a developmental factor, unc-62, to already long-lived worms increases lifespan and healthspan. Biology open. 2017:bio. 027433. 10.1242/bio.027433 PMC5769649 29055022

[44] Ni H, Xu M, Zhan GL, Fan Y, Zhou H, Jiang HY, et al The GWAS Risk Genes for Depression May Be Actively Involved in Alzheimer's Disease. Journal of Alzheimer's disease: JAD. 2018;64(4):1149–61. Epub 2018/07/17. 10.3233/JAD-180276 . 30010129

[45] Sekar S, McDonald J, Cuyugan L, Aldrich J, Kurdoglu A, Adkins J, et al Alzheimer's disease is associated with altered expression of genes involved in immune response and mitochondrial processes in astrocytes. Neurobiology of aging. 2015;36(2):583–91. Epub 2014/12/03. 10.1016/j.neurobiolaging.2014.09.027 25448601 PMC4315763

[46] Acquaah-Mensah GK, Agu N, Khan T, Gardner A. A regulatory role for the insulin- and BDNF-linked RORA in the hippocampus: implications for Alzheimer's disease. Journal of Alzheimer's disease: JAD. 2015;44(3):827–38. Epub 2014/11/02. 10.3233/JAD-141731 . 25362032

[47] Kummer MP, Hammerschmidt T, Martinez A, Terwel D, Eichele G, Witten A, et al Ear2 deletion causes early memory and learning deficits in APP/PS1 mice. The Journal of neuroscience: the official journal of the Society for Neuroscience. 2014;34(26):8845–54. Epub 2014/06/27. 10.1523/jneurosci.4027-13.2014 24966384 PMC4147626

[48] Cavallini A, Brewerton S, Bell A, Sargent S, Glover S, Hardy C, et al An unbiased approach to identifying tau kinases that phosphorylate tau at sites associated with Alzheimer's disease. Journal of Biological Chemistry. 2013:jbc. M113. 463984. 10.1074/jbc.M113.463984 PMC3743503 23798682

[49] Lin H, Zhang T, Wu Y, Wang Y, Wang W, Wang Q. Related Genes and Potential Biomarkers for Early Diagnosis of Alzheimer’s Disease: A Preliminary Study Based on DNA Microarray. American Journal of Alzheimer's Disease & Other Dementias. 2014;29(1):90–5. 10.1177/1533317513506774 PMC11008138 24381135

[50] Donev R, Newall A, Thome J, Sheer D. A role for SC35 and hnRNPA1 in the determination of amyloid precursor protein isoforms. Molecular psychiatry. 2007;12(7):681 10.1038/sj.mp.4001971 17353911 PMC2684093

[51] Chiocco MJ, Zhu X, Walther D, Pletnikova O, Troncoso JC, Uhl GR, et al Fine mapping of calcineurin (PPP3CA) gene reveals novel alternative splicing patterns, association of 5′ UTR trinucleotide repeat with addiction vulnerability, and differential isoform expression in Alzheimer's disease. Substance use & misuse. 2010;45(11):1809–26. 20590401 10.3109/10826084.2010.482449 PMC3031160

[52] Schöbel S, Neumann S, Hertweck M, Dislich B, Kuhn P-H, Kremmer E, et al A novel sorting nexin modulates endocytic trafficking and α-secretase cleavage of the amyloid precursor protein. Journal of Biological Chemistry. 2008;283(21):14257–68. 10.1074/jbc.M801531200 18353773

[53] Treusch S, Hamamichi S, Goodman JL, Matlack KE, Chung CY, Baru V, et al Functional links between Abeta toxicity, endocytic trafficking, and Alzheimer's disease risk factors in yeast. Science (New York, NY). 2011;334(6060):1241–5. Epub 2011/10/29. 10.1126/science.1213210 22033521 PMC3281757

[54] Schobel S, Neumann S, Hertweck M, Dislich B, Kuhn PH, Kremmer E, et al A novel sorting nexin modulates endocytic trafficking and alpha-secretase cleavage of the amyloid precursor protein. The Journal of biological chemistry. 2008;283(21):14257–68. Epub 2008/03/21. 10.1074/jbc.M801531200 . 18353773

[55] Zhu L, Zhong M, Zhao J, Rhee H, Caesar I, Knight EM, et al Reduction of synaptojanin 1 accelerates Aβ clearance and attenuates cognitive deterioration in an Alzheimer mouse model. The Journal of biological chemistry. 2013;288(44):32050–63. Epub 2013/09/21. 10.1074/jbc.M113.504365 24052255 PMC3814799

[56] Kim J-R, Lee S-R, Chung HJ, Kim S, Baek S-H, Kim JH, et al Identification of amyloid β-peptide responsive genes by cDNA microarray technology: Involvement of RTP801 in amyloid β-peptide toxicity. Experimental & Molecular Medicine. 2003;35(5):403–11. 10.1038/emm.2003.53 14646594

[57] Alkallas R, Fish L, Goodarzi H, Najafabadi HS. Inference of RNA decay rate from transcriptional profiling highlights the regulatory programs of Alzheimer's disease. Nat Commun. 2017;8(1):909–. 10.1038/s41467-017-00867-z . 29030541 PMC5714957

[58] Ikiz B, Alvarez MJ, Ré DB, Le Verche V, Politi K, Lotti F, et al The Regulatory Machinery of Neurodegeneration in In Vitro Models of Amyotrophic Lateral Sclerosis. Cell Rep. 2015;12(2):335–45. Epub 07/02. 10.1016/j.celrep.2015.06.019 . 26146077 PMC4646662

[59] Tseveleki V, Rubio R, Vamvakas S-S, White J, Taoufik E, Petit E, et al Comparative gene expression analysis in mouse models for multiple sclerosis, Alzheimer's disease and stroke for identifying commonly regulated and disease-specific gene changes. Genomics. 2010;96(2):82–91. Epub 05/07. 10.1016/j.ygeno.2010.04.004 . 20435134 PMC4205236

[60] Boyle AP, Araya CL, Brdlik C, Cayting P, Cheng C, Cheng Y, et al Comparative analysis of regulatory information and circuits across distant species. Nature. 2014;512(7515):453 10.1038/nature13668 25164757 PMC4336544

