# Supplementary figures and images for: Caenorhabditis elegans hub genes that respond to amyloid beta are homologs of genes involved in human Alzheimer’s disease
Source: PLoS One. 2019 Jul 10;14(7):e0219486. doi: 10.1371/journal.pone.0219486 (PMC6619800; doi:10.1371/journal.pone.0219486)

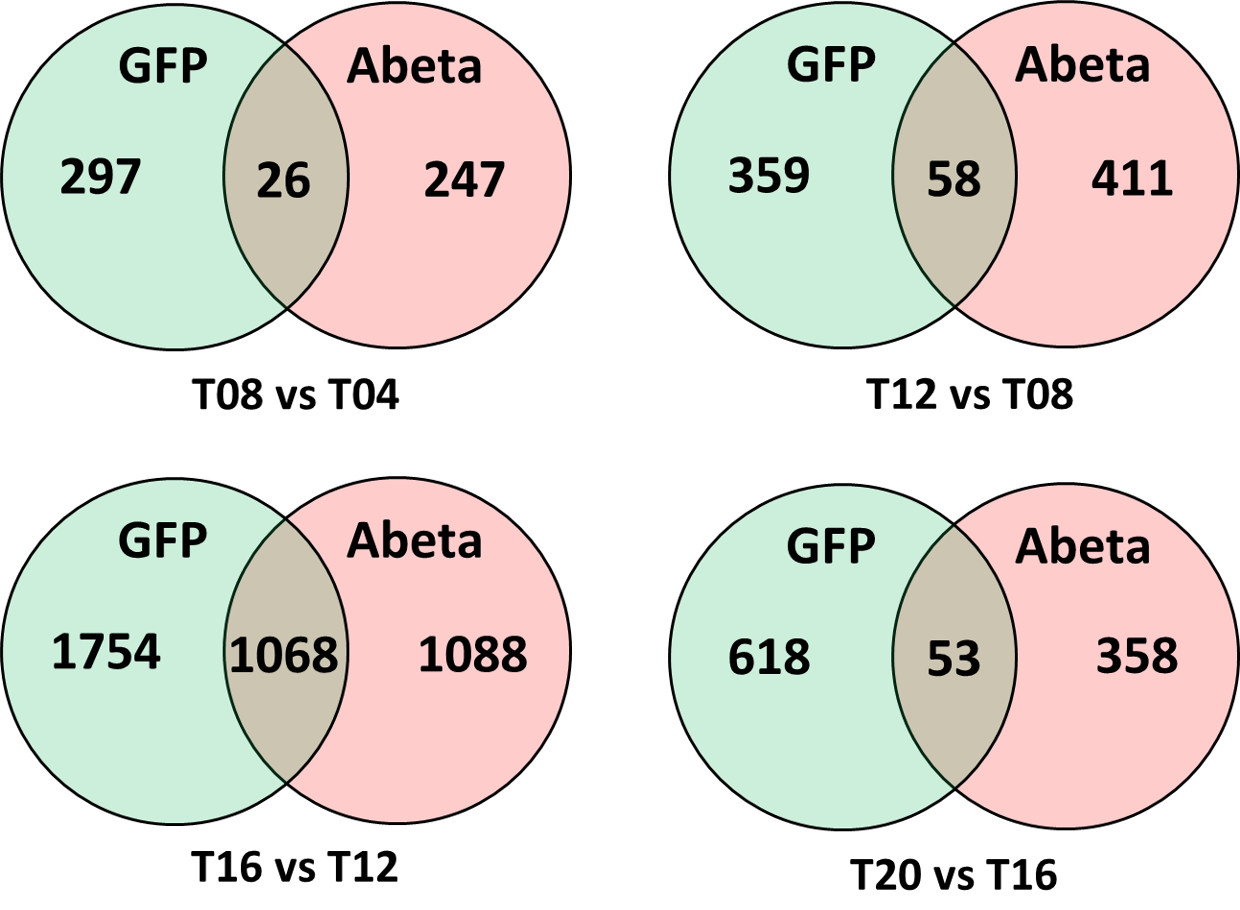

Supplement: S1 Fig — (TIF) [file pone.0219486.s001.tif]

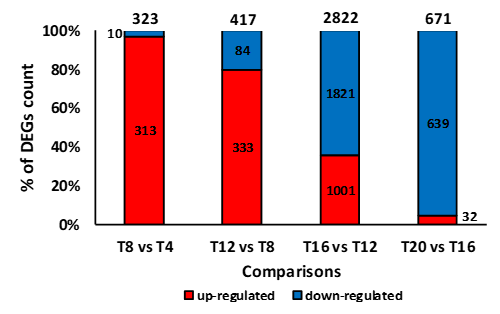

Supplement: S2 Fig — (TIF) [file pone.0219486.s002.tif]

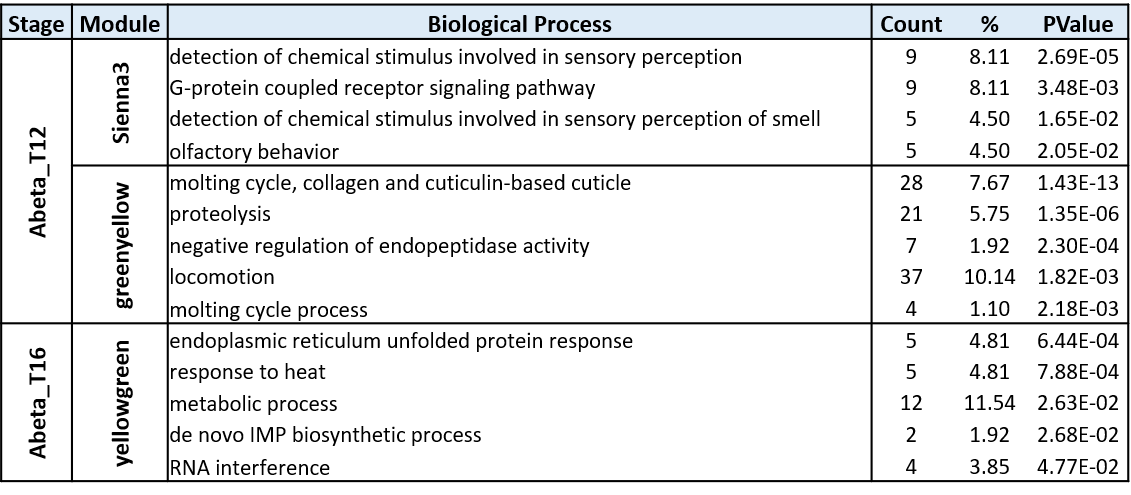

Supplement: S3 Fig — Three unique modules were detected for Abeta responsive gene list (Fig 8) were subjected to GO analysis. (TIF) [file pone.0219486.s003.tif]

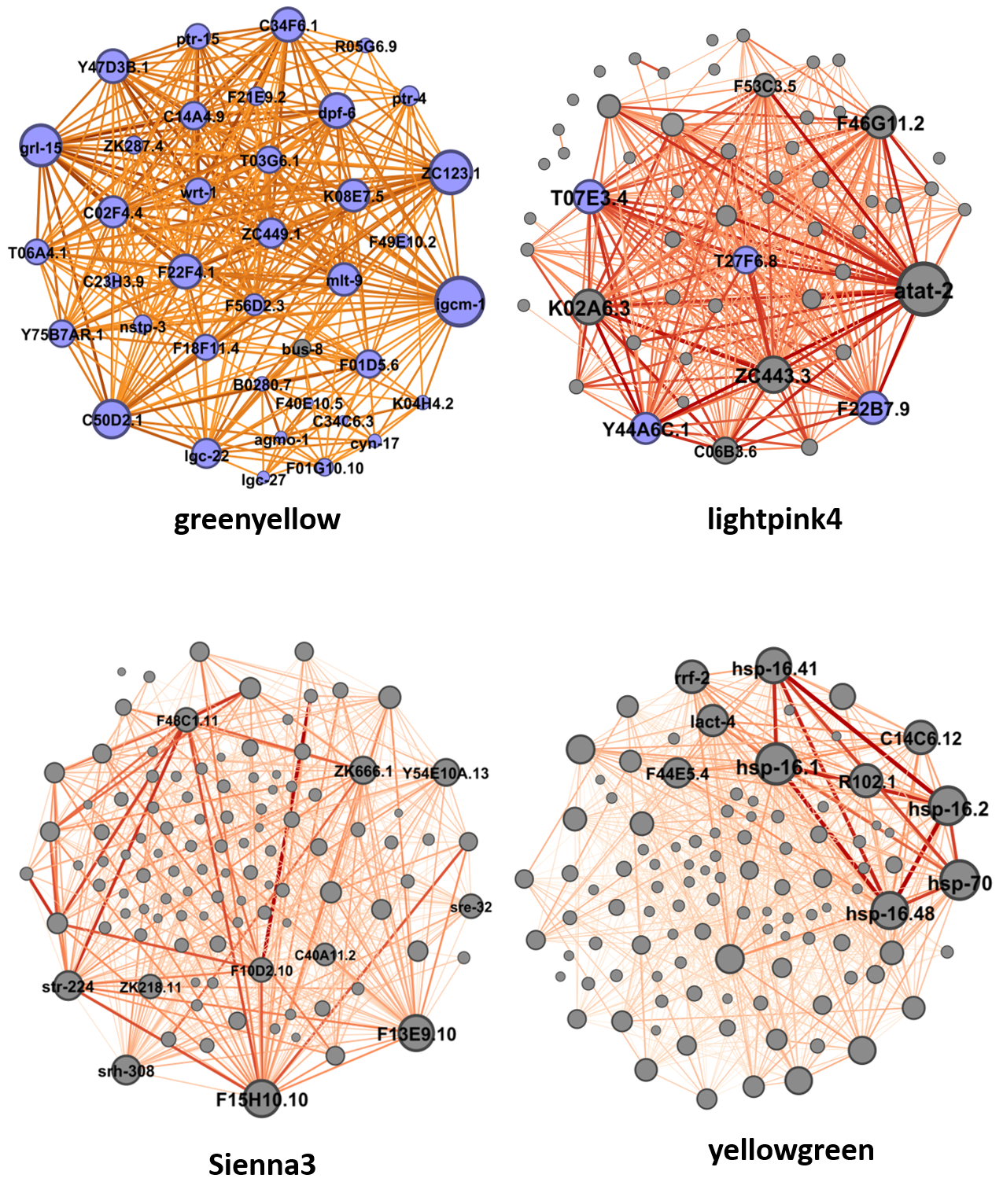

Supplement: S4 Fig — The analysis was included in Figs 9 and 10 in the main text. (TIF) [file pone.0219486.s004.tif]
